# Sequential activation of Notch and Grainyhead gives apoptotic competence to Abdominal-B expressing larval neuroblasts in *Drosophila* Central nervous system

**DOI:** 10.1371/journal.pgen.1008976

**Published:** 2020-08-31

**Authors:** Asif Bakshi, Rashmi Sipani, Neha Ghosh, Rohit Joshi

**Affiliations:** 1 Laboratory of Drosophila Neural Development, Centre for DNA Fingerprinting and Diagnostics (CDFD), Inner Ring Road, Uppal, Hyderabad, India; 2 Graduate Studies, Manipal Academy of Higher Education, Manipal, India; New York University, UNITED STATES

## Abstract

Neural circuitry for mating and reproduction resides within the terminal segments of central nervous system (CNS) which express Hox paralogous group 9–13 (in vertebrates) or Abdominal-B (Abd-B) in *Drosophila*. Terminal neuroblasts (NBs) in A8-A10 segments of *Drosophila* larval CNS are subdivided into two groups based on expression of transcription factor Doublesex (Dsx). While the sex specific fate of Dsx-positive NBs is well investigated, the fate of Dsx-negative NBs is not known so far. Our studies with Dsx-negative NBs suggests that these cells, like their abdominal counterparts (in A3-A7 segments) use Hox, Grainyhead (Grh) and Notch to undergo cell death during larval development. This cell death also happens by transcriptionally activating *RHG* family of apoptotic genes through a common apoptotic enhancer in early to mid L3 stages. However, unlike abdominal NBs (in A3-A7 segments) which use increasing levels of resident Hox factor Abdominal-A (Abd-A) as an apoptosis trigger, Dsx-negative NBs (in A8-A10 segments) keep the levels of resident Hox factor Abd-B constant. These cells instead utilize increasing levels of the temporal transcription factor Grh and a rise in Notch activity to gain apoptotic competence. Biochemical and *in vivo* analysis suggest that Abdominal-A and Grh binding motifs in the common apoptotic enhancer also function as Abdominal-B and Grh binding motifs and maintains the enhancer activity in A8-A10 NBs. Finally, the deletion of this enhancer by the CRISPR-Cas9 method blocks the apoptosis of Dsx-negative NBs. These results highlight the fact that Hox dependent NB apoptosis in abdominal and terminal regions utilizes common molecular players (Hox, Grh and Notch), but seems to have evolved different molecular strategies to pattern CNS.

## Author summary

Two major characteristic features of bilaterian organisms are the head to tail axis and a complex central nervous system. The Hox family of transcription factors, which are expressed segmentally along the head to tail axis, plays a critical role in determining both of these features. One of the ways by which Hox factors do this is by mediating differential programmed cell death of the neural stem cells along the head to tail axis of the developing central nervous system, thereby regulating the numerical diversity of the neurons generated along this axis. Our study with a subpopulation of neural stem cells in the most terminal region of the *Drosophila* larval central nervous system highlights that region-specific Hox-dependent cell death of neural stem cells in abdominal and terminal regions utilizes common molecular players (Hox, Grh and Notch), but seems to have evolved different molecular strategies to pattern the developing central nervous system.

## Introduction

Establishment of precise neural circuitry is a prerequisite for a functional central nervous system (CNS). This precision critically relies on generation of right cellular diversity (and numbers) in a region specific manner across the anterior-posterior (AP) axis of developing CNS [1, 2]. Coordination of quiescence, proliferation and differentiation of neural stem cells (NSCs) is critical for generating the cellular diversity in CNS [3]. An alternative but less common mode used to regulate cell numbers in developing CNS is the apoptosis of NSC itself [4–13]. While apoptosis of neuronal and glial cells plays an important role in establishing a functional neural circuitry, the death of the NSCs is expected to bring about gross changes to the developing CNS [9, 14]. Hox genes specify the AP axis of the developing CNS, and also play an important role in apoptosis of both NSCs as well their progeny [4, 5, 8–13, 15–24]. However, the molecular mechanism of Hox mediated NSC apoptosis in different regions of the developing CNS is yet to be elucidated.

Generation of cellular diversity also depends on integration of spatial identity of the NSC with temporal series transcription factors (tTFs) [1, 25] and different signalling pathways. One such pathway is Notch signalling, which is utilized for making binary cell fate choice and plays divergent and reiterative roles in different cellular contexts in developing CNS [26]. Specific cellular context for Notch signalling is expected to depend on combinatorial binding of spatial and temporal transcription factors with CSL proteins (CBF1/RBP-J, Su(H), Lag-1), thereby causing differential target gene activation [26–28]. Consequently, this is expected to result in different cell fates in the same spatial domain or same cell fates in different spatial domains. For example, reiterative and context specific roles of Notch in tandem with tTFs has been shown to coordinate both survival and apoptosis of neurons within a single lineage of developing CNS [29]. However, how Notch signalling in combination with spatial and tTFs generates cellular diversity in different regions of CNS is not completely understood [26]

**Fig 1:**
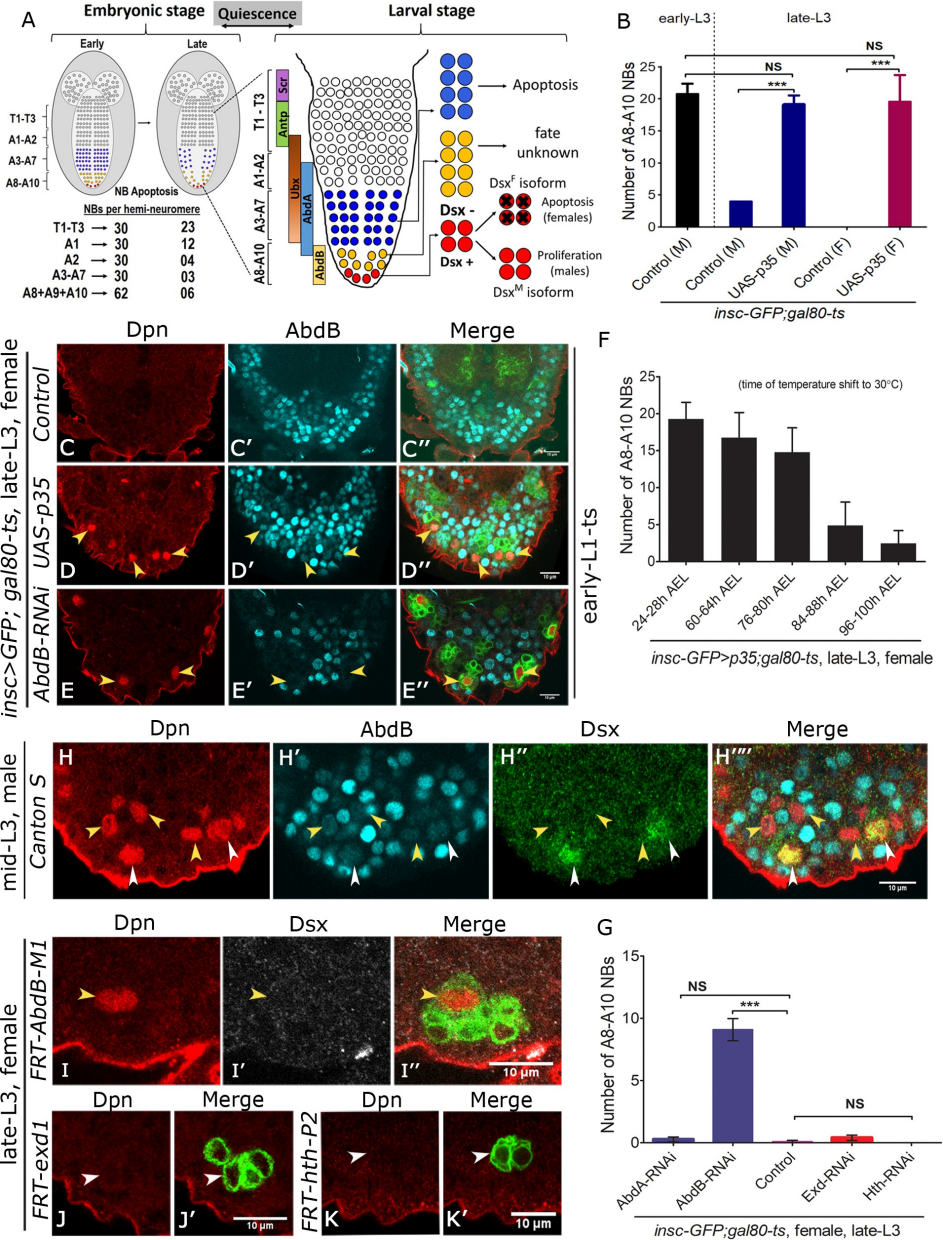
Dsx-negative NBs undergo Abd-B mediated apoptosis independent of canonical cofactors Exd and Hth. (**A**) Schematic of embryonic CNS showing that NBs in T1-T3 and A1-A10 segments undergo apoptosis in late embryonic stages. Number of NBs in these hemineuromeres before and after the apoptosis are summarized. Following embryonic apoptosis NBs undergo quiescence and start dividing again in early larval stages. Within larval VNC thoracic NBs (white cells in T1-A2 segments), abdominal NBs (blue cells in A3-A7 segments) and terminal NBs (yellow cells in A8-A10 segments) are shown. In terminal (A8-A10) segments, four out of twelve NBs express Dsx (shown in red and referred to as Dsx-positive NBs) which continue dividing through late L3 stages in males and undergo apoptosis in mid L2 stage in females. The fate of remaining Dsx-negative NBs (shown in yellow) is unknown. (**B**) Comparison of A8-A10 NBs in control male VNCs (m) in early L3 stage versus control and p35 expressing male (m) and female (f) VNCs in late L3 stage. (**C-E**) Show that control female VNC does not have any A8-A10 NBs (marked by anti-Dpn) in late L3 stage (**C**) while the expression of anti-apoptotic gene p35 (**D**) or knockdown of Abd-B by RNAi (**E**) (TS, S1A Fig) results in ectopic A8-A10 NBs. (**F**) Quantification of total number of surviving NBs observed in A8-A10 segments of female VNCs resulting from induction of p35 expression at different developmental times (indicated as hours AEL along the x-axis). (**G**) Comparison of A8-A10 NBs in late L3 stage in female VNCs of control versus RNAi mediated knockdown for Abd-A, Abd-B, Exd and Hth. (**H**) Male VNC showing that Dsx-negative NBs express the resident Hox gene Abd-B prior to their apoptosis in mid L3 stage. (**I-K)** MARCM analysis shows that Dsx-negative NBs mutant for *Abd-B*^*M1*^ do not undergo apoptosis (**I**), but die normally in case of *exd*^*1*^ (**J**) and *hth*^*P2*^ mutants (**K**). GFP marked *exd*^*1*^ and *hth*^*P2*^ mutant lineages lacking the NB are shown (by white arrowheads). Yellow arrowheads indicate Dsx-negative NBs. Scale bars are 10μm. All the images are single confocal sections. In current and subsequent figures sex of the dissected larvae is mentioned on each figure panel. Graph shows mean±s.d. Significance (*P*-value) is from two-tailed Student's unpaired *t*-test.

In *Drosophila*, NSCs (also called neuroblasts-NBs) undergo Hox dependent apoptosis in both embryonic as well as larval stages [4, 5, 7–13] by activation of *RHG* family of apoptotic genes [30–33]. In embryonic CNS approximately 60 NBs (30 per hemineuromere) are reported in each of T1-A7 (T1-T3 and A1-A7) segments. While the segments A8, A9 and A10 together have 124 NBs (62 NBs collectively in all three hemineuromeres of A8, A9 and A10) [4, 14, 34, 35]. These NBs undergo Hox dependent apoptosis in late embryonic stages leaving behind 46 NBs each (23 NBs per hemineuromere) in T1-T3; 24 NBs (12 NBs per hemineuromere) in A1; 8 NBs (4 NBs per hemineuromere) in A2; 6 NBs each (3 NBs per hemineuromere) in A3-A7; and 12 NBs (6 NBs collectively in 3 hemineuromeres) in total across A8, A9 and A10 [4, 14, 34, 35]. Subsequently these NBs enter quiescence and start dividing again in early larval stages (Fig 1A).

In larval CNS, Hox dependent apoptosis of postembryonic NBs has been reported in Labial [8], Deformed, Sex combs reduced (Scr) [10], Abdominal-A (Abd-A) [5] and Abdominal-B (Abd-B) [9] expressing regions. Of these abdominal NB apoptosis (in A3-A7 segments) in larval ventral nerve cord (VNC) occurs in response to a temporal increase in expression of Abd-A [5]. This apoptosis relies on TFs Extradenticle (Exd) and Grainyhead (Grh) as well as Notch signalling [12]. Together these factors activate *RHG* genes *grim* and *reaper* through a 717bp apoptotic enhancer [12]. This enhancer lies within a 22Kb genomic region called neuroblast regulatory region (NBRR [36]), which was identified by using overlapping genomic deletions *MM3* and *XR38* on Chromosome-3L [36].

Terminal region of larval VNC (A8-A10 segments) expresses Hox factor Abd-B (Fig 1A) and harbours neurons which form the neural circuitry central for adult mating behaviour [37–42]. In embryonic stages, the terminal region has 124 NBs (62 across 3 hemineuromeres). Majority of these NBs undergo apoptosis in late embryonic stage, while the remaining 12 cells enter quiescence [4, 9, 34] (Fig 1A). These NBs exit quiescence in early larval stages and are referred to as terminal NBs (hereon referred to as A8-A10 NBs) [14, 43]. Four out of these 12 NBs express *doublesex* gene (*dsx*) and are referred to as Dsx-positive NBs while the remaining 8 are referred to as Dsx-negative NBs [14, 43]. Dsx-positive NBs undergo sex-specific apoptosis in early larval stages in females [9] using Abd-B and female specific isoform of Dsx (Dsx^F^) [13]. Abd-B and Dsx^F^ (but not Dsx^M^-male specific isoform of Dsx) cooperatively interact on motifs of 717bp apoptotic enhancer causing the activation of RHG genes *grim* and *reaper*. This female specific apoptosis of Dsx-positive NBs has been shown to be independent of Grh and Notch [13]. However, the fate of Dsx-negative NBs cells is not known thus far [9, 14, 43] (Fig 1A).

In this body of work, we show that Dsx-negative NBs, like their abdominal counterparts (in A3-A7 segments), undergo Hox dependent apoptosis. This apoptosis also happens in the mid third instar larval (mid L3) stage of development and is mediated by activation of *RHG* genes (*grim* and *reaper*), through a 717bp abdominal apoptotic enhancer. But in contrast to abdominal segments where NB apoptosis relies on increasing levels of Hox factor Abd-A [5], we find that Dsx-negative NBs keep the levels of resident Hox factor Abd-B constant across larval stages. These NBs instead utilize an increase in levels of temporal series transcription factor Grh and rise in Notch activity to undergo apoptosis. The ligand Delta for the activation of Notch signalling seems to reside only on its neuronal progeny and not on glia as previously suggested [11]. Moreover, we show that increasing Grh expression and Notch activity is important in determining the apoptotic competence of these NBs. We find that this apoptosis occurs independent of Hox cofactors Exd and Homothorax (Hth). Biochemical experiments show that Abd-B physically interacts with Grh. Subsequent *in vitro* and *in vivo* studies indicate that DNA motifs bound by Abd-A-Grh in the abdominal region [12] also function as Abd-B-Grh binding motifs in the terminal region for maintaining the activity of the enhancer in Dsx-negative NBs. Congruent to this, we find that deletion of the apoptotic enhancer by the CRISPR-Cas9 method results in a block of Dsx-negative NB apoptosis. These results underline the fact that segment specific Hox dependent NB apoptosis utilizes overlapping molecular players, but seems to have evolved different molecular strategies to pattern CNS.

## Results

### Dsx-negative NBs undergo Abd-B mediated apoptosis independent of Exd and Hth

Previous literature suggests that out of twelve NBs in the terminal region of larval CNS [14, 43], four Dsx-positive NBs undergo sex-specific apoptosis in females (in mid L2 stage) while their male counterparts continue to divide till late L3 stages [9, 13]. The fate of Dsx-negative NBs however has not been investigated (Fig 1A). We started out by counting the number of NBs (Dpn expressing cells) in Abd-B expressing region of the male VNCs in early L3 stage. We could count as many as 20 A8-A10 NBs in this region (20.75+/- 1.58; n = 9 VNCs, N = 2, Fig 1B, bar-1). Since this number includes 4 Dsx-positive NBs as well, we conclude that the number of Dsx-negative NBs in CNS at this stage is approximately 16, which is different from what has been reported earlier [43]. Subsequently in late L3 stage, the number of A8-A10 NBs surviving in this region dropped to 4 Dsx-positive NBs in case of male VNCs and zero in case of female VNCs (Fig 1B, bar-2 and 4; Fig 1C–1C”). In order to check if Dsx-negative NBs also undergo apoptosis, we expressed cell death blocker p35 in NBs from early L1 stage using temporally inducible GAL4 system (*tub-GAL80*^*ts*^*; insc-GAL4*-used in Temperature Shift experiments; hereon referred to as TS; shown in S1A Fig). In comparison to wild type controls, we observed that expression of p35 resulted in survival of NBs in late L3 stages for both female (19.5+/- 4.2; n = 14 VNCs, N = 4, Fig 1B, bar-5 and Fig 1D) and male VNCs (19.07+/-1.4, n = 13 VNCs, N = 3, Fig 1B, bar-3). Since the number of surviving NBs in both cases are more than 4 (Fig 1B), we concluded that remaining NBs would be Dsx-negative. Costaining of p35 expressing female VNC (in late L3 stage) with both Dpn and Dsx further confirmed that Dsx-negative NBs indeed undergo apoptosis (S2D Fig). To conclusively establish this, we checked female VNCs in mid L2 and mid L3 stages by costaining them with apoptotic markers Dcp-1/Casp-3 and NB marker Dpn (S2A–S2C Fig). We could observe Dcp-1/Casp-3 staining in terminal NBs in mid L3 stages, indicating that Dsx-negative NBs indeed undergo apoptosis (S2B and S2C Fig).

Next, we identified the temporal window of Dsx-negative NB apoptosis by inducing p35 expression in NBs of female VNCs at different developmental stages (S1 Data). Our results suggested that Dsx-negative NBs start undergoing apoptosis in the early L3 stage and die asynchronously over a period of next 48 hrs with majority of death occurring around mid L3 stage (Fig 1F; TS, S1A–S1E Fig). This is similar to what has been reported for abdominal NBs in A3-A7 segments [5].

Abdominal NBs are known to undergo apoptosis in response to a pulse of Abd-A in early to mid L3 stages [5]. Therefore, we wanted to check whether Dsx-negative NBs also express the resident Hox factor Abd-B prior to their apoptosis. We observed that Dsx-negative NBs costained for Abd-B in mid L3 stage (yellow arrowheads in Fig 1H’). Next, we tested the functional importance of Abd-B expression in these cells. This was done by knocking down Abd-B by RNA interference (RNAi) as well as by making MARCM clones for Abd-B null allele (*Abd-B*^*M1*^). RNAi for Abd-B was induced in female VNCs from early L1 stage (TS, S1A Fig) and we could recover as many as 9 A8-A10 NBs in late L3 stage (9.1+/- 2.9, n = 11 VNCs, N = 3, Fig 1G, bar-2 and yellow arrowheads in Fig 1D–1D”), while control VNCs did not show any NBs at the same stage (Fig 1G, bar-3, Fig 1C–1C”). Since these NBs would include 4 Dsx-positive NBs as well, therefore we costained these VNCs with Dsx in addition to Dpn and were able to observe Dsx-negative NBs surviving in late L3 stage (S2E Fig). Similarly, inducing MARCM clones for *Abd-B*^*M1*^ null allele resulted in recovery of clones with surviving Dsx-negative NBs in late L3 stage (yellow arrowheads in Fig 1I–1I”) (n = 10 clones were recovered from 16 VNCs). Abd-A and Abd-B have an overlapping expression in VNC. In order to rule out the role of Abd-A in apoptosis of Dsx-negative NBs, we knocked down Abd-A protein by RNAi from early L1 stage (TS, S1A Fig) and checked for surviving Abd-B positive NBs in terminal region of female VNC in late L3 stage. Though we could recover abdominal NBs in A3-A7 segments of VNC (S2F and S2G Fig), we could not recover any NBs positive for Abd-B in terminal segments (Fig 1G, bar-1). This suggested that Abd-A is not involved in apoptosis of Abd-B expressing NBs.

Hox genes are known to carry out *in vivo* functions with help of their cofactors Exd and Hth. We had earlier shown that in case of abdominal NBs apoptosis, Abd-A requires its cofactor Exd but not Hth [12]. Therefore, we tested the requirement of both of these cofactors in Dsx-negative NB apoptosis by RNAi mediated knockdown as well as by making MARCM clones for *exd*^*1*^ and *hth*^*P2*^ alleles. Induction of RNAi for Exd and Hth from early L1 stage (TS, S1A Fig) did not result in any surviving NBs in the terminal region of CNS (Fig 1G, bar-4 and 5). Similarly, induction of MARCM clones from early L1 to mid L3 stage for both *exd*^*1*^ null allele and for a strong hypomorph of *hth* (*hth*^*P2*^), did not result in any ectopic NBs in late L3 stages (Fig 1J and 1K, 15 and 14 VNCs were analyzed for *exd*^*1*^ and *hth*^*P2*^ respectively). In both cases, we could recover remnants of GFP marked lineages but no associated NBs. This suggested that NBs mutant for Exd or Hth divided a few times before normally undergoing apoptosis (white arrowheads in Fig 1J and 1K). Therefore, unlike abdominal NBs which use Exd as a Hox cofactor neither Exd nor Hth is required for Dsx-negative NB apoptosis.

Together these results show that Dsx-negative NBs undergo apoptosis in the same temporal window (early to mid L3 stages) as that of abdominal NBs located in A3-A7 segments. These results also suggest that while abdominal NBs rely on both Hox and Exd, Dsx-negative NBs apoptosis relies only on Abd-B and is independent of the canonical Hox cofactors like Exd and Hth.

### Grh and Notch are required for apoptosis of Dsx-negative NBs

**Fig 2:**
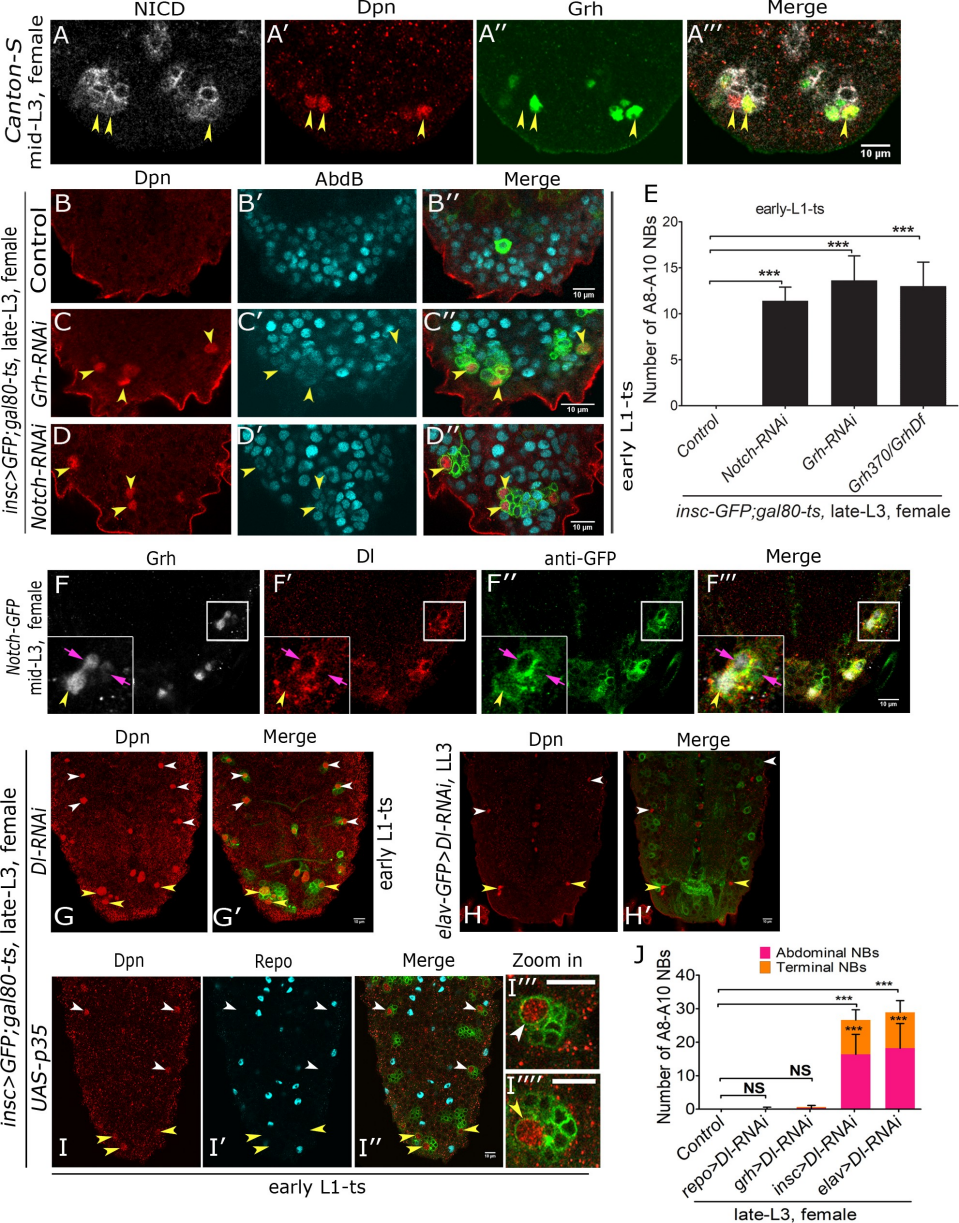
Grh and Notch are required for Dsx-negative NB apoptosis. (**A**) Shows Dsx-negative NBs expressing NICD and Grh in mid L3 stage female VNC. (**B-D**) Compared to control (**B**), knock down of Grh (**C**) and Notch (**D**) (TS, S1A Fig) show a block of A8-A10 NB apoptosis in late L3 female VNC. (**E**) Quantification of the A8-A10 NBs across various genotypes in late L3 stage female VNCs is shown. (**F)** Shows expression of Notch ligand Delta in NB, GMC and neuronal progeny in a lineage marked by Notch-GFP in female VNC at mid L3 stage. Inset shows NB lineage indicated by square. (**G-H**) Knockdown of Notch ligand Delta in NBs (**G**) and neurons (**H**) results in a block of abdominal and Dsx-negative NB apoptosis in female VNC in late L3 stage. (**I**) Abdominal and terminal NB lineages where NB apoptosis has been blocked by p35 expression (TS, S1A Fig) do not show any glial cells even in late L3 stage. (**I”’ and I”“**) Shows a magnified view of abdominal and terminal NB lineages. (**J**) Graph comparing the number of surviving NBs in female VNCs in late L3 stage when *Delta-RNAi* knockdown was done in NBs, GMC, glia and neurons in both abdominal and terminal regions. Dsx-positive NBs undergo apoptosis in mid L2 stage in female VNCs, this death occurs independent of Notch and Grh, therefore A8-A10 NBs in all the panels (except panel “I”) will be Dsx-negative. Yellow arrowheads indicate Dsx-negative NBs, white arrowheads indicate abdominal NBs, pink arrows indicate GMC in panel “F”. Scale bars are 10μm. All the images are single confocal sections. Graph shows mean±s.d. Significance (*P*-value) is from two-tailed Student's unpaired *t*-test.

Since abdominal NBs require both Grh and Notch signalling to undergo apoptosis [12], we tested their expression and requirement in Dsx-negative NB apoptosis. As Dsx-positive NBs die in mid L2 stage, only surviving cells in A8-A10 segments at mid L3 stage will be Dsx-negative NBs. We observed that both Grh and Notch Intracellular Domain (NICD) are expressed in Dsx-negative NBs at mid L3 stage (yellow arrowheads in Fig 2A–2A”’). Next, we tested the functional requirement of *grh* and *Notch* gene in Dsx-negative NB apoptosis. Since neither of these genes are required for death of Dsx-positive NB apoptosis in females [13], therefore any surviving NB in the terminal region will be Dsx-negative. The RNAi knockdown *for grh* and *Notch* was induced from early L1 stage of development (TS, S1A Fig). This resulted in survival of approximately 13 Dsx-negative NBs (13.63+/-2.65, n = 11 VNCs, N = 3) in case of *grh-RNAi* (yellow arrowheads in Fig 2C; Fig 2E, bar-3) and 12 Dsx-negative NBs (11.41+/-1.48, n = 15 VNCs, N = 4) in case of *Notch-RNAi* (yellow arrowheads in Fig 2D; Fig 2E, bar-2). The *grh-RNAi* result corroborated with the number of the Dsx-negative NBs recovered in late L3 stage for CNS specific *grh* mutant allelic combination (*grh*^*370/Df*^) (13+/- 2.61, n = 8 VNCs, N = 3) (Fig 2E, bar-4).

Next, we wanted to establish the source of the ligand for the activation of Notch signalling in these NBs. In case of abdominal NBs, it has been suggested, that ligand Delta expressed in progeny glia or neurons possibly signals the activation of Notch in NB [11]. We tested the expression of Notch and its ligand Delta in Dsx-negative NB lineages in female VNCs at mid L3 stage. For this we used the Notch protein trap line (Notch-GFP, BDSC-81271) to mark NB lineages. We found that Delta was expressed in NBs as well as in GMC, both of which were marked by Grh expression (yellow and pink arrows in Fig 2F). Delta expression was also seen in the nearby cells which did not express Grh. Subsequently, we found that Delta was expressed in NBs and associated lineages in early L2, late L2 and early L3 stages (S3A–S3D Fig). Its expression in abdominal and terminal segments reduced significantly by late L3 stage (S3E Fig), by when all of the NBs in the region have undergone apoptosis. Knockdown for *Delta* gene in NBs (using *inscGAL4*) from early L1 stage (TS, S1A Fig) in female VNCs resulted in block of NB apoptosis in both abdominal (16.38+/- 5.9, n = 13 VNCs, N = 3) and terminal regions (10.23+/- 3.05, n = 13 VNCs, N = 3) (white and yellow arrowheads respectively, Fig 2G and 2J bar-4). Considering that *inscGAL4* shows strong expression in NB as well as its progeny, we used *grhGAL4* and *elavGAL4* for the knockdown of the *Delta* gene. Former expresses mainly in GMC and NB, while the latter shows a weak expression in NBs but a strong expression in post-mitotic neurons. While the knockdown of *Delta* in *elavGAL4* domain blocked NB apoptosis in both abdominal (18.2+/- 7.3, n = 11 VNCs, N = 3) and terminal regions (10.7+/- 3.49, n = 11 VNCs, N = 3) (Fig 2H and 2J, bar-5), *grhGAL4* was unable to do so (Fig 2J, bar-3). This was surprising as GMC is closer to NB than neurons. To rule out expression inconsistency, we tested if *grhGAL4* could recapitulate the expression of endogenous Grh in the larval stages. We found that *grhGAL4* driven nuc-GFP coexpressed with Grh protein in NBs from early L2 to mid L3 stages (S4A–S4D Fig). Therefore, it is most likely that the low expression levels of the *grhGAL4* in NBs and GMC may be the reason for its inability to block apoptosis in case of Delta knockdown.

A previous report had suggested that in a lineage, progeny glia can act as the source of Delta ligand [11] for the mother NB. Therefore, we tested the presence of repo-positive glial cells in these lineages by blocking the apoptosis of NBs by p35 expression from early L1 stage (TS, S1A Fig). We did not find any repo-positive cells in terminal (Fig 2I”“) or abdominal (Fig 2I”’) NB lineages (212 lineages were checked in n = 7 VNCs, N = 2). In order to rule out the role of cortex glia in NB apoptosis we knocked down *Delta* from early L1 stage (TS, S1A Fig) using *repoGAL4* line in female VNCs. We did not observe any block of terminal or abdominal NB apoptosis (Fig 2J, bar-2) in this case as well. In order to exclude the potency issue, the glial knockdowns were done from early embryonic stages using two different GAL4 lines (BDSC-7415, [44]), yet we could not get any significant block of NB apoptosis in terminal or abdominal regions.

These results suggest that like abdominal NBs, Dsx-negative NBs need both Grh and Notch signalling to undergo Abd-B mediated apoptosis. The source of Notch ligand Delta is either NB itself or its progeny neurons but not glial cells which are born out of the same lineage; nor the pre-existing embryonic glial cells present in the VNC.

### Enhancer responsible for Dsx-negative NBs apoptosis resides within 14.5kb region of NBRR

**Fig 3:**
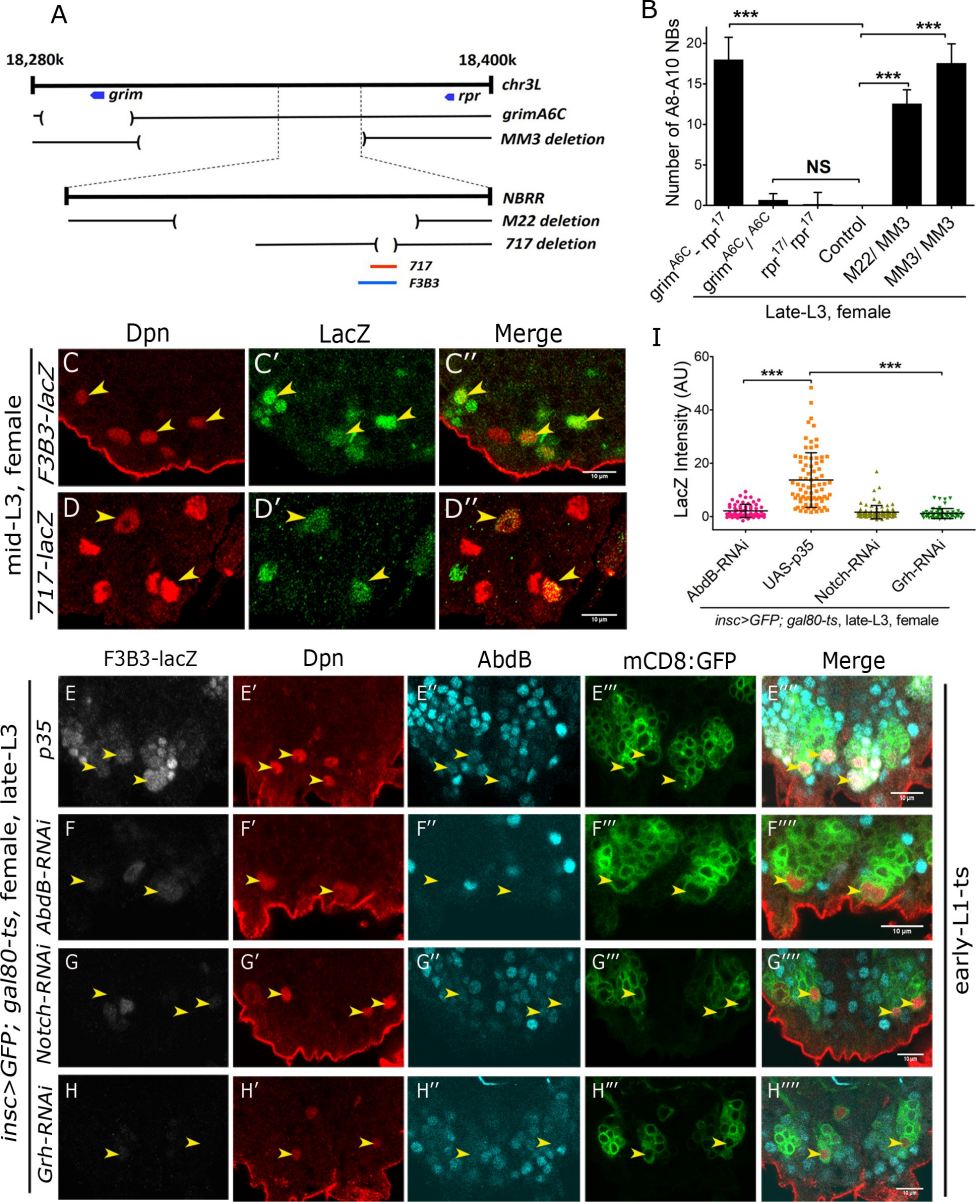
Abdominal apoptotic enhancer is responsive to Abd-B, Grh and Notch in Dsx-negative NBs. (**A**) Schematic showing the genomic region surrounding NBRR. Approximate extent of genomic deletions tested for narrowing down the location of apoptotic enhancer are shown. Extent and location of 1Kb (*F3B3-blue line*) and 717bp enhancers (*red line*) are also indicated. (**B**) Graph comparing total number of A8-A10 NBs observed (in female VNCs) across different genotypes at late L3 stage. (**C-D**) *Enhancer-lacZ* for 1Kb abdominal apoptotic enhancer (*F3B3*) and its 717 bp subfragment show expression in Dsx-negative NBs prior to their apoptosis in mid L3 stage female VNC. (**E-H**) Comparison of *F3B3-lacZ* intensity in A8-A10 NBs of p35 expressing female VNC versus VNCs with *Abd-B*, *grh* and *Notch* knockdown (TS, S1A Fig). (**I**) Graph comparing *F3B3-lacZ* intensity in A8-A10 NBs in female VNCs expressing p35 versus VNCs with *Abd-B*, *Notch* and *grh* knockdown (TS, S1A Fig). 20% of A8-A10 NBs quantified in case of *UAS-p35* and *Abd-B-RNAi* will be Dsx-positive while rest 80% will be Dsx-negative. Yellow arrowheads indicate A8-A10 NBs. Scale bars are 10μm. All the images are single confocal sections. Graph shows mean±s.d. Significance (*P*-value) is from one way ANNOVA test and two-tailed Student's unpaired *t*-test.

The enhancer responsible for the apoptosis of abdominal NBs had been narrowed down to 1kb region (and its 717 bp sub-fragment) [12] of 22 kb NBRR of the genome [36] (Fig 3A). We wanted to test if the same enhancer could be responsible for the apoptosis of Dsx-negative NBs. We started out by using a combination of genomic deletions *MM3* (which uncovers entire NBRR and surrounding regions, [36]) and *M22* (which deletes a 14.5 Kb genomic region of NBRR, [12]) to test for the block of apoptosis of Dsx-negative NBs. We could recover as many as 18 NBs (17.57+/- 2.37; n = 14 VNCs, N = 2) in late L3 stages in female VNC for *MM3/MM3* homozygotes (Fig 3B, bar-6) and 13 NBs (12.57+/- 1.69; n = 19 VNCs, N = 2) in case of *M22/MM3* trans-heterozygotic combination (Fig 3B, bar-5 and yellow arrowheads in S4G Fig), both of which included 4 Dsx-positive NBs as well. These results suggested that enhancer responsible for the apoptosis of Dsx-negative NBs also resided within the 14.5kb region of the genome. Expectedly like in case of abdominal NBs, we found that Dsx-negative NBs also relied on both *grim* and *reaper* genes for their apoptosis (yellow arrowheads in S4F Fig). The deletion of either of the gene alone (*grim*; Fig 3B, bar-2 and *reaper*; Fig 3B, bar-3) did not show any significant block in death of Dsx-negative NBs. Subsequently, we tested and found that *enhancer-lacZ* lines made from 1Kb (*F3B3-lacZ*) (Fig 3C) and its 717bp sub-fragment (*717-lacZ*) (Fig 3D and S4H–S4K Fig) express in Dsx-negative NBs at mid L3 stage prior to their death. We also observed that the lacZ expression showed a significant increase in NBs prior to their apoptosis (S4K Fig). Next, we used RNAi to knockdown *grh*, *Abd-B* and *Notch* in A8-A10 NBs and scored for their effect on 1Kb *F3B3-lacZ* reporter line expression. The knockdown was induced from early L1 stage and larvae were dissected in late L3 stage (TS, S1A Fig). The effect of these knockdowns on *F3B3-lacZ* in A8-A10 NBs was quantitated and compared to lacZ levels in A8-A10 NBs that have been blocked for apoptosis by expression of p35. We found *F3B3-lacZ* expression was consistently downregulated in case of *grh-RNAi* (n = 60 NBs, Average intensity = 1.10+/-1.88), *Abd-B-RNAi* (n = 63 NBs, Average intensity = 2.09+/-2.49) and *Notch-RNAi* (n = 92 NBs, Average intensity = 1.63+/- 2.51) (Fig 3E–3H), as compared to NBs expressing p35 (n = 80 NBs, Average intensity = 13.67+/-10.26) (Fig 3E–3E”“and 3I). In case of Grh and Notch, which do not have a role in Dsx-positive NB apoptosis, all the surviving NBs will be Dsx-negative. But, in case of p35 and *Abd-B-RNAi*, 20% of total number of cells are expected to be Dsx-positive NBs, which cannot be detected owing to technical limitation of four confocal channels. However, the majority of remaining cells (80%) will be Dsx-negative, and therefore we conclude that apoptotic *enhancer-lacZ* is responsive to Abd-B, Grh and Notch in Dsx-negative NBs.

Collectively, these results establish that Dsx-negative NBs, like their abdominal counterparts, undergo apoptosis. This apoptosis requires activation of both *grim* and *reaper* through 1Kb abdominal apoptotic enhancer, which is activated by Grh, Notch and resident Hox gene (Abd-B).

### Deletion of 657 bp of the apoptotic enhancer is sufficient to block Dsx-negative NB cell death

**Fig 4:**
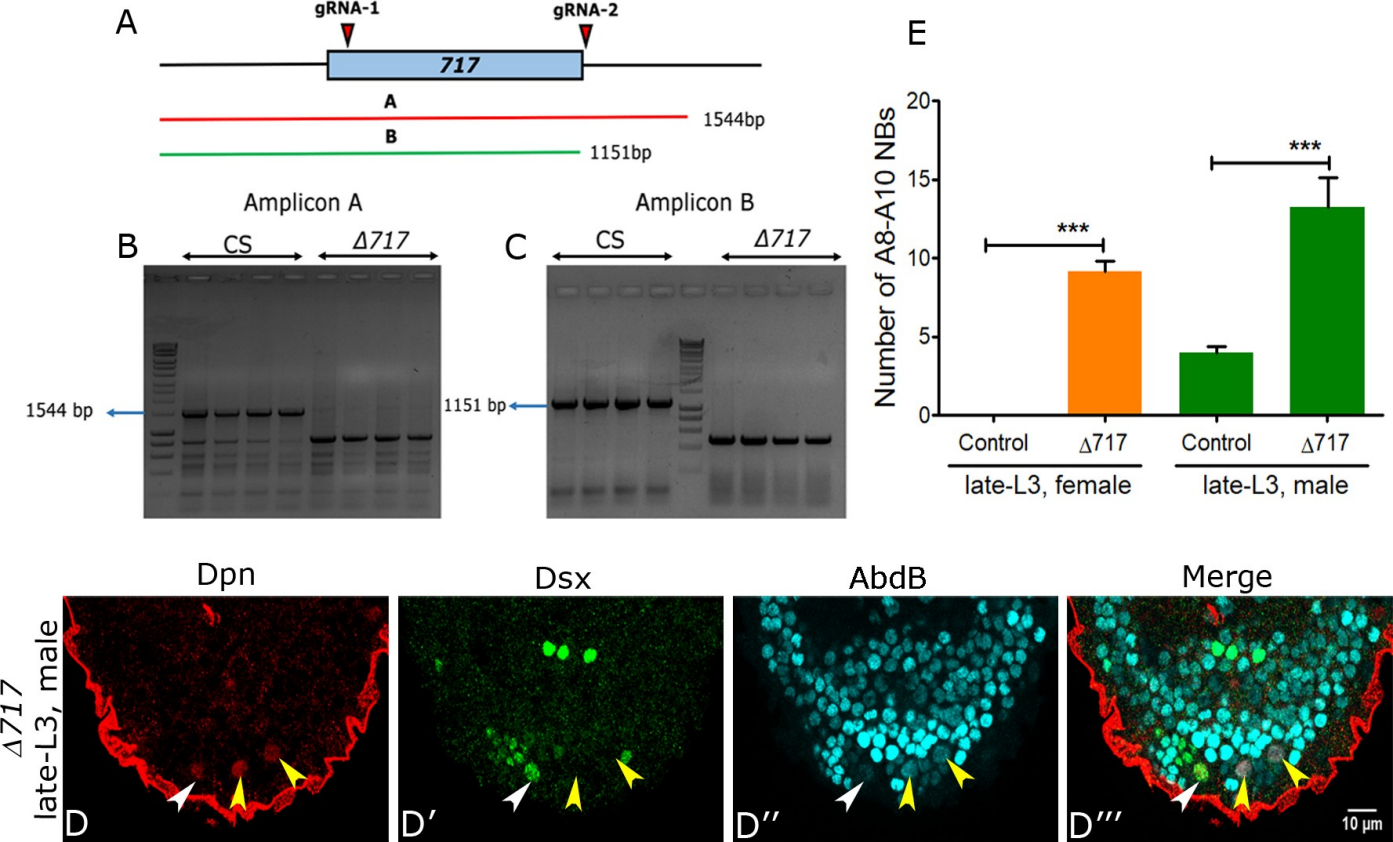
Deletion of 659 bp region of 1Kb enhancer blocks Dsx-negative NB apoptosis. (**A**) Shows the approximate position of the two *guide-RNAs* w.r.t. highly conserved 717bp sub-fragment of the 1Kb apoptotic enhancer. The approximate extent of the two PCR amplicons used to detect genomic deletion are also shown. (**B-C**) Gels showing the genomic PCR for the control (*Canton-S*) and homozygous larvae (for amplicons A and B) confirming the enhancer deletion. (**D**) Shows Dsx-negative NBs surviving in male VNC at late L3 stage. (**E**) Graph comparing the number of surviving A8-A10 NBs in the terminal region of female and male VNCs at late L3 stage. Yellow and white arrowheads indicate Dsx-negative and Dsx-positive NBs respectively. Scale bar is 10μm. Single confocal section is shown in “D”. Graph shows mean±s.d. Significance (*P*-value) is from two-tailed Student's unpaired *t*-test.

In order to conclusively test if 1kb *F3B3* played a role in the death of the Dsx-negative NBs, we targeted highly conserved 717 bp sub-fragment of 1Kb region using CRISPR-Cas9 method (Fig 4A). We could delete 659 bp of the 717 bp region leaving 58 bp in 5’ region of the enhancer intact as verified by genomic PCR for two overlapping amplicons in the region (Fig 4B and 4C). Deletion was tested for survival of Abd-B expressing Dsx-negative NBs in homozygous mutant larvae in late L3 stage. We could recover approximately 9 NBs (9.1+/-0.7; n = 14 VNCs, N = 3) in female VNCs (Fig 4E) and 14 NBs (13.2+/- 1.9; n = 14 VNCs, N = 3) in male VNCs (Fig 4D and 4E) compared to female and male control larvae which have zero and 4 NBs (Dsx-positive NBs) respectively in late L3 stage. This result shows that that 717bp fragment of 1Kb *F3B3* enhancer is indeed relevant for the apoptosis of Dsx-negative NBs.

### Abd-B binds to Abd-A binding sites on the enhancer

One of the characteristic features of an apoptotic enhancer is its ability to maintain its expression till the target cells undergo apoptosis. In case of abdominal NBs, we had identified 8 DNA motifs responsible for the maintenance of the apoptotic enhancer activity till late larval stages [12]. Since Dsx-negative NBs initiate apoptosis in mid L3 stage, and use Hox, Grh and Notch signalling like in case of abdominal NBs, we checked the binding of Abd-B and Grh on the maintenance motifs of the abdominal apoptotic enhancer.

Three out of these eight maintenance motifs (motifs-27,30 and 32 shown in S5I Fig) had shown a complex formation for Abd-A-Exd-Grh on the DNA [12]. Since Exd is not required for apoptosis of A8-A10 NBs, we tested Abd-B and Grh binding on these motifs by EMSA. We found that all the motifs bound Abd-B and Grh individually (S5A–S5H Fig). Except for one (motif-33) (black arrowhead, lane-65 and 66 in S5F Fig), none of the motifs exhibited a trimer complex of DNA-Abd-B-Grh (S5A–S5E Fig, S5G–S5H Fig). This indicated that unlike Abd-A which along with Exd and Grh exhibited higher order complex formation (DNA-Abd-A-Exd-Grh or DNA-Abd-A-Grh) [12], Abd-B did not show any such higher order complex formation with Grh. The specificity of Abd-B binding on these motifs was checked by testing the oligonucleotides mutant for AT-rich sequences (S5A–S5H Fig). These AT-rich sequences had been shown to bind Abd-A-Exd by EMSA earlier [12]. Grh binding sites and their specificity of binding on these motifs had also been tested previously [12]. These results suggest that AT-rich binding sites on the enhancer were capable of functioning as both Abd-A or Abd-B binding sites to possibly enable the use of apoptotic enhancer in two different regions of developing CNS.

### Maintenance activity of the apoptotic enhancer in Dsx-negative NBs relies on Abd-B, Grh and Su(H) binding sites

**Fig 5:**
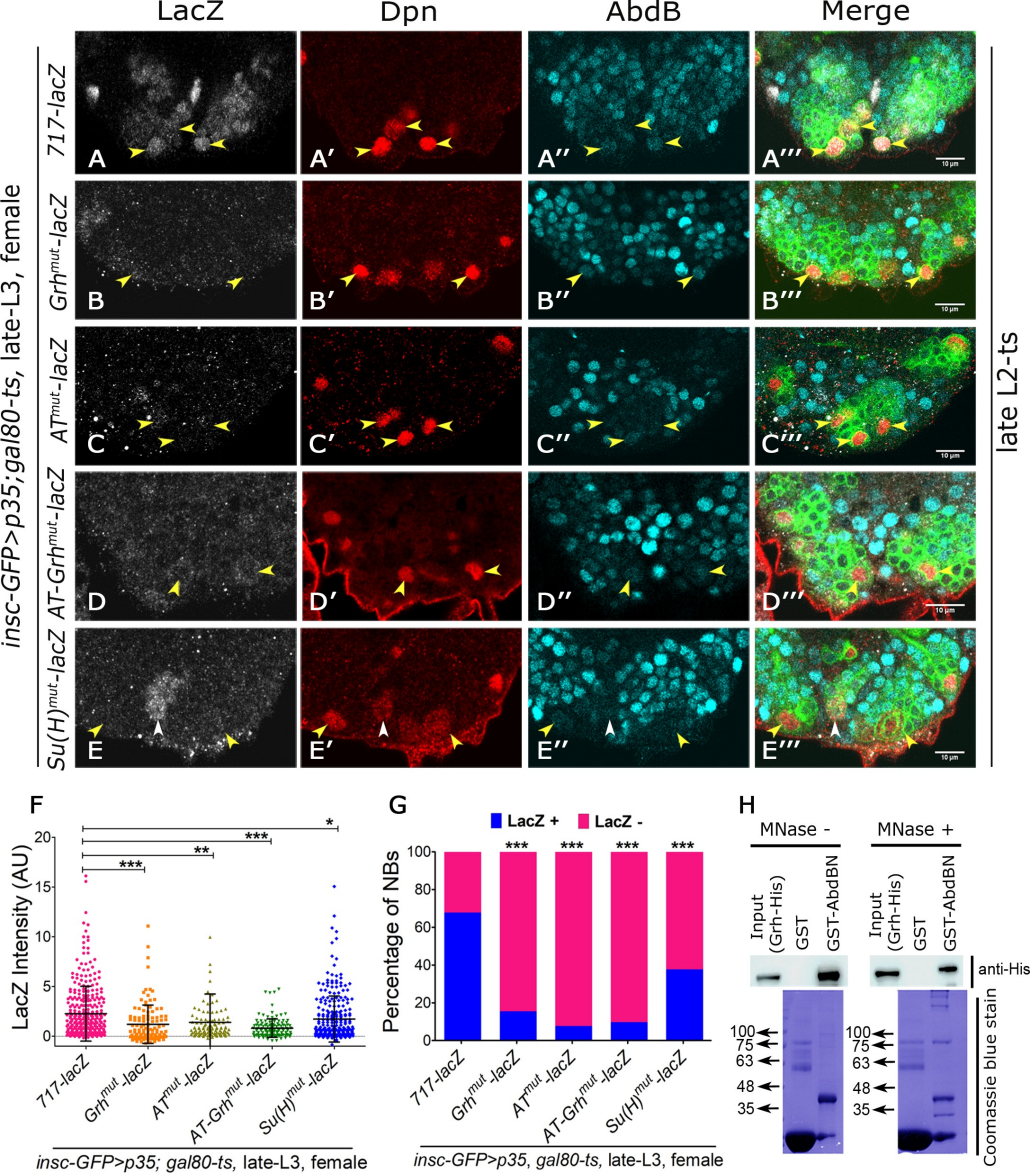
Hox, Grh and Su(H) binding sites used in abdominal segments are also responsible for maintaining the activity of enhancer in Dsx-negative NBs. (**A-E**) Show the *enhancer-lacZ* expression in Dsx-negative NBs for the wild type and various mutant versions of the enhancer in p35 expressing female VNCs at late L3 stage. It is observed that *717-lacZ* expresses normally in Dsx-negative NBs (**A**), but *Grh*^*mut*^*-lacZ* (**B**), *AT*^*mut*^*-lacZ* (**C**) and *AT-Grh*^*mut*^*-lacZ* (**D**) fail to maintain their expression. (**E**) *Su(H)*^*mut*^*-lacZ* is partly compromised in its ability to maintain its expression and a fraction of Dsx-negative NBs show normal lacZ expression (white arrowhead in E). (**F**) Graph showing a significant decrease of *lacZ* intensity for different mutagenized versions of the enhancer compared to wild type enhancer in Dsx-negative NBs of female VNCs at late L3 stage. (**G**) Graph showing a decrease in percentage of *lacZ* expressing cells in different mutagenized versions of the enhancer compared to wild type enhancer in Dsx-negative NBs of female VNCs at late L3 stage. (**H**) Western blot showing that GST-Abd-B is capable of pulling down His-tagged Grh from bacterial lysate while GST alone is unable to do so. This Abd-B-Grh interaction is stable even in presence of micrococcal nuclease treatment. Coomassie Blue depicts almost equal loading of the GST-tagged protein samples. Yellow arrowheads indicate Dsx-negative NBs. White arrowheads indicate a fraction of Dsx-negative NBs (observed only in case of *Su(H)*^*mut*^*-lacZ*) where lacZ reporter expression could still be detected. Scale bars are 10μm. All images are single confocal sections. Graph shows mean±s.d. Significance (*P*-value) is from one way ANNOVA test and two-tailed Student's unpaired *t*-test.

In order to test the *in vivo* role of Abd-B-Grh binding motifs in enhancer maintenance activity in Dsx-negative NBs, we checked the expression of various mutagenized versions of the *enhancer* (detailed below and schematized in S6A Fig). The first construct had Grh binding sites mutagenized across all the 8 maintenance motifs present in 717 bp enhancer, leaving AT rich binding sites largely intact (*Grh*^*mutant*^*-lacZ*; [12]). In the second construct, all AT rich binding sites were mutagenized (*AT*^*mutant*^*-lacZ*) across eight maintenance motifs. The third construct tested was mutant for both AT-rich binding sites and Grh binding sites (*AT-Grh*^*mutant*^*-lacZ;* also referred to as *HEG*^*mutant*^*-lacZ* previously [12]). Fourth construct was used to test the role of Notch signalling in abdominal NB apoptosis and had Suppressor of Hairless (Su(H)) binding sites mutagenized in 717 bp enhancer (*Su(H)*^*mutant*^*-lacZ;* [12]). The maintenance activity of wild type and mutagenized versions of the enhancer were compared in Dsx-negative NBs of female VNCs at late L3 stage. This was achieved by blocking the apoptosis of these cells using expression of p35 from late L2 stage (TS, S1B Fig), by when the Dsx-positive NBs have already undergone apoptosis in female VNCs. We observed that while *717-lacZ* maintained its expression in late L3 stage, *Grh*^*mutant*^*-lacZ*, *AT*^*mutant*^*-lacZ* and *AT-Grh*^*mutant*^*-lacZ* could not maintain the enhancer activity in Dsx-negative NBs in late L3 stage (yellow arrowheads in Fig 5A–5D, and Fig 5F and 5G). Interestingly in case of *Su(H)*^*mutant*^*-lacZ* we found that while a significant percentage of Dsx-negative NBs were unable to maintain the expression of lacZ in late L3 stage (yellow arrowheads in Fig 5E), approximately 37% of these NBs did show some visible expression of the lacZ in VNC (white arrowheads in Fig 5E). A mean fluorescence intensity quantitation for lacZ expression in Dsx-negative NBs expressing p35 was compared across different *enhancer-lacZ* lines (Fig 5F). We observed that *Grh*^*mutant*^*-lacZ* (1.2 +/- 1.93; n = 12 VNCs, N = 3), *AT*^*mutant*^*-lacZ* (1.38+/- 2.85; n = 11 VNCs, N = 3), *AT-Grh*^*mutant*^*-lacZ* (0.81+/- 0.93; n = 11 VNCs, N = 3), and *Su(H)*^*mutant*^*-lacZ* (1.72 +/- 2.3; n = 10 VNCs, N = 4) show a significant decrease in lacZ expression compared to *717-lacZ* (2.26+/- 2.75; n = 16 VNCs, N = 5) (Fig 5F). A quantitation of the percentage of lacZ expressing Dsx-negative NBs at late L3 stage also showed a significant decrease for the mutant *enhancer-lacZ* lines compared to control *717-lacZ* (Fig 5G). In case of *Su(H)*^*mutant*^*-lacZ*, we found that 37% of cells showed lacZ expression in NBs. We believe that this is due to additional, yet to be identified, Su(H) binding sites which are important for enhancer maintenance. However, considering a significant decrease in fluorescence intensity quantitation and reduction in percentage of NBs expressing *enhancer-lacZ*, we conclude that like Abd-B and Grh, Su(H) binding sites mutagenized in the enhancer also have a role in its activity maintenance.

Our previous results show that Abd-A and Grh interact with each other *in vitro* [12]. The *enhancer-lacZ* results suggest that Abd-A-Grh motifs are also utilized as Abd-B-Grh binding motifs *in vivo* (S5 Fig and Fig 5A–5G). Therefore, we decided to test *in vitro* interaction of Abd-B and Grh by GST-pulldown assay. To this end, bacterially expressed GST tagged Abd-B and GST alone were tested for their interaction with His tagged Grh. We found that while GST alone could not pulldown Grh, GST-Abd-B was successful in pulling down His-Grh *in vitro* (Fig 5H). This interaction remained intact in the presence of micrococcal nuclease, thereby ruling out the possibility that bacterial DNA in the lysate facilitated the interaction of these two proteins.

These results indicate that Abd-B and Grh are capable of interacting with each other in vitro. Results also indicate that Abd-A binding sites found in eight maintenance motifs of abdominal apoptotic enhancer are capable of functioning as Abd-B binding sites *in vivo*. The *enhancer-lacZ* data further suggests that Hox, Grh and Su(H) binding sites together play an important role in maintaining activity of the enhancer in Dsx-negative NBs. Though in case of Su(H), it is likely that additional binding sites which are yet to be identified, may also play a role in enhancer maintenance.

### Increase in Grh expression and Notch activity precedes the death of Dsx-negative NBs

**Fig 6:**
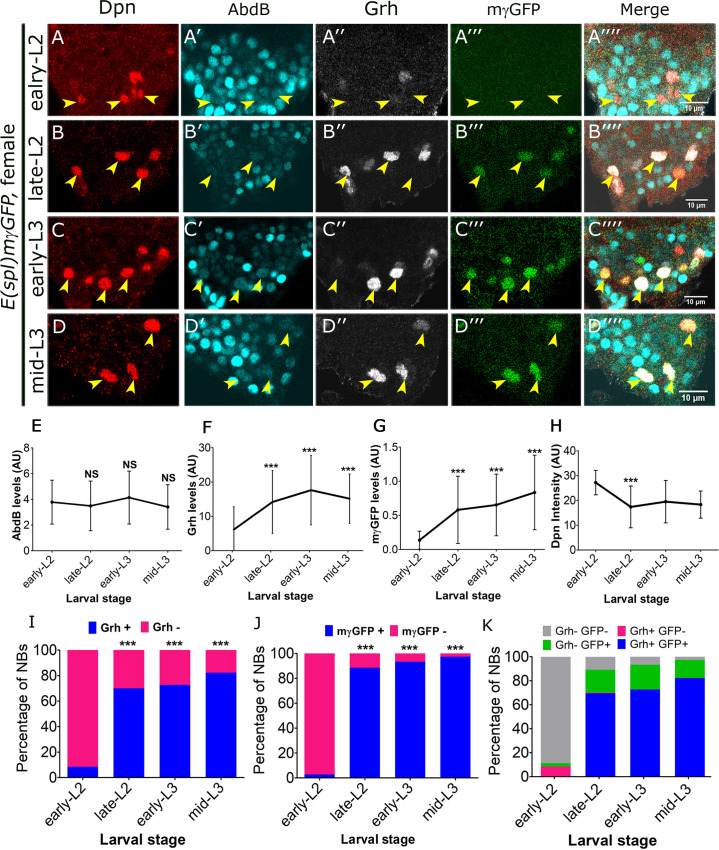
Increase in Notch activity and Grh expression precedes the death of Dsx-negative NBs. (**A-D**) Shows an increase in Grh and E(spl)mγ-GFP expression and near constant Abd-B expression across early L2 (**A**), late L2 (**B**), early L3 (**C**) and mid L3 stages (**D**) in Dsx-negative NBs. (**E-H**) Graphs showing quantitation of Abd-B (**E**), Grh (**F**), E(spl)mγ-GFP (**G**) and Dpn (**H**) intensities in Dsx-negative NBs across different stages. (**I** and **J**) Graphs showing an increase in percentage of Grh expressing (**I**) and E(spl)mγ-GFP expressing (**J**) Dsx-negative NBs in female larval VNCs across different stages. (**K**) Graph comparing the percentage of different populations of NBs based on their Grh and E(spl)mγ-GFP expression status across different stages of development. Graph shows that increase in Notch activity precedes Grh expression in Dsx-negative NBs as E(spl)mγ-GFP and Grh expressing NBs show a significant increase from early L2 to late L2 stage. Yellow arrowheads indicate both Dsx-positive and Dsx-negative NBs in panel “A” and Dsx-negative NBs in panels “B-D”. Scale bars are 10μm. All images are single confocal sections. Graph shows mean±s.d. Significance (*P*-value) is from two-tailed Student's paired *t*-test.

Even though we could observe the expression of Abd-B in terminal NBs in early L2 stage, we noticed that these cells undergo apoptosis only by early-mid L3 stage. Since, we did not observe any qualitative difference in the levels of Abd-B in terminal NBs across these stages, we carried out a quantitative comparison of the expression of Abd-B, Grh and Notch signalling in female CNS across different stages; early L2 (52–56 hrs AEL; Fig 6A–6A”“), late L2 (68–72 hrs AEL; Fig 6B–6B”“); early L3 (80–84 hrs AEL; Fig 6C–6C”“) and mid L3 (92–96 hrs AEL; Fig 6D–6D”“). Dsx costaining was not possible in this experiment owing to the technical limitation of four confocal channels. However, since we analysed female VNCs, NBs in all these stages will be Dsx-negative NBs, except for early L2 stage wherein there will be a mixed population with 20% of the cells being Dsx-positive. E(spl)mγ-GFP was used to monitor Notch signalling in these cells as it shows a strong expression and is known to be responsive to the Notch pathway in NBs [45]. We observed that while the levels of Abd-B in Dsx-negative NBs remained mostly constant across different stages (Fig 6A’–6D’ and Fig 6E), it was the levels of Grh (Fig 6A”–6D” and Fig 6F) and E(spl)mγ-GFP (Fig 6A”’–6D”’ and Fig 6G) which showed a significant temporal increase in these cells from early L2 to mid L3 stage. There was also an increase in the total number of cells that started expressing Grh and E(spl)mγ-GFP in the late L2 stage (Fig 6I and 6J) as compared to the early L2 stage. The increase in expression preceded the temporal window when Dsx-negative NBs start undergoing apoptosis. Dpn levels were quantified in these cells, which served as a control (Fig 6H). Additionally, we examined the relative abundance of different cell populations across different stages. We observed that in the early L2 stage, majority of NBs were Grh^-^ GFP^-^ (~89%, gray bar), and ~8% of NBs were Grh^+^ GFP^-^, and only ~3% of the NBs were Grh^-^ GFP^+^. In subsequent stages, we observed a significant increase in Grh^-^ GFP^+^ and Grh^+^ GFP^+^ cells, but we did not see any increase in Grh^+^ GFP^-^ cells, and this trend continued in later stages as well. This analysis indicated that NBs sequentially activate Notch followed by Grh, resulting in the transition of the cells from Grh^-^ GFP^-^>Grh^-^ GFP^+^ >Grh^+^ GFP^+^ state (Fig 6K). These results ruled out the role of Hox gene Abd-B and suggested that increase in activity of Notch and expression of Grh act as a trigger for the apoptosis of A8-A10 NBs. This observation is in contrast to abdominal NBs where a pulse of Abd-A expression is responsible for the cell death [5].

A parallel comparison of the levels of Grh and E(spl)mγ-GFP in Abd-A expressing abdominal NBs in the same VNCs indicated that the levels of Grh as well as Notch activity did not show a significant increment in abdominal cells across these stages (S7A–S7G Fig). In abdominal NBs, Grh has been reported to sustain the Abd-A expression during late larval stages to maintain *RHG* expression, thereby facilitating apoptosis [7]. In A8-A10 NBs we do not see any significant increase in levels of Hox gene Abd-B and instead we observe an increase in levels of Grh. However, we still tested if Grh could be playing a similar role in maintaining stable expression of Abd-B or vice versa. We observed that in A8-A10 NBs, Grh and Abd-B did not cross regulate each other (S1 Data, S8L–S8N Fig and S8G and S8H Fig and S8J Fig). On similar lines, we also tested and found that Notch and Abd-B did not cross regulate each other in these cells (S1 Data, S8D–S8F Fig and S8G, S8I and S8J Fig).

Collectively our results highlight the fundamental differences between the apoptotic mechanism of NBs in the abdominal versus terminal region of CNS. In these regions we find overlapping players (Hox, Grh and Notch) employing different molecular strategies to cause region specific NB apoptosis (through a common apoptotic enhancer). These results also underline the capacity of qualitatively different Notch signalling activities to integrate divergent molecular inputs in different regions of VNC [abdominal segments with increasing Abd-A and constant Grh versus terminal segments with increasing Grh and constant Abd-B], leading to a common end result of NB apoptosis.

### Notch and Grh are important for apoptotic competence of Dsx-negative NBs

Existing literature implicates the role of Notch and Grh in determining the competence of the NBs [7, 46–49]. Prior to testing their role in determining the apoptotic competence of the NBs, we tested Grh and Notch for any possible cross regulation. We found that these two genes did not cross regulate each other in A8-A10 NBs (S1 Data, S8A–S8C Fig and S8G, S8I and S8K Fig).

In order to test the individual roles of Grh and Notch in determining the apoptotic competence of Dsx-negative NBs, we decided to knockdown either of them individually in the background of Abd-B overexpression. To this end, it is important to note that in A8-A10 segments, Abd-B plays a role in apoptosis of Dsx-negative NBs (Fig 1I), as well as sex-specific apoptosis of Dsx-positive NBs in females [13]. Apoptotic potential of Abd-B is also highlighted by its capacity to cause apoptosis of thoracic NBs as well [13]. Since Abd-B induces Dsx expression in NBs [13], its apoptotic property in females is potentiated by Dsx^F^ and resisted in males by Dsx^M^-which is known to block NB apoptosis [13]. Expectedly we also found that Abd-B overexpression in Dsx-positive NBs is incapable of causing their apoptosis in male VNCs (S9A and S9B Fig).

**Fig 7:**
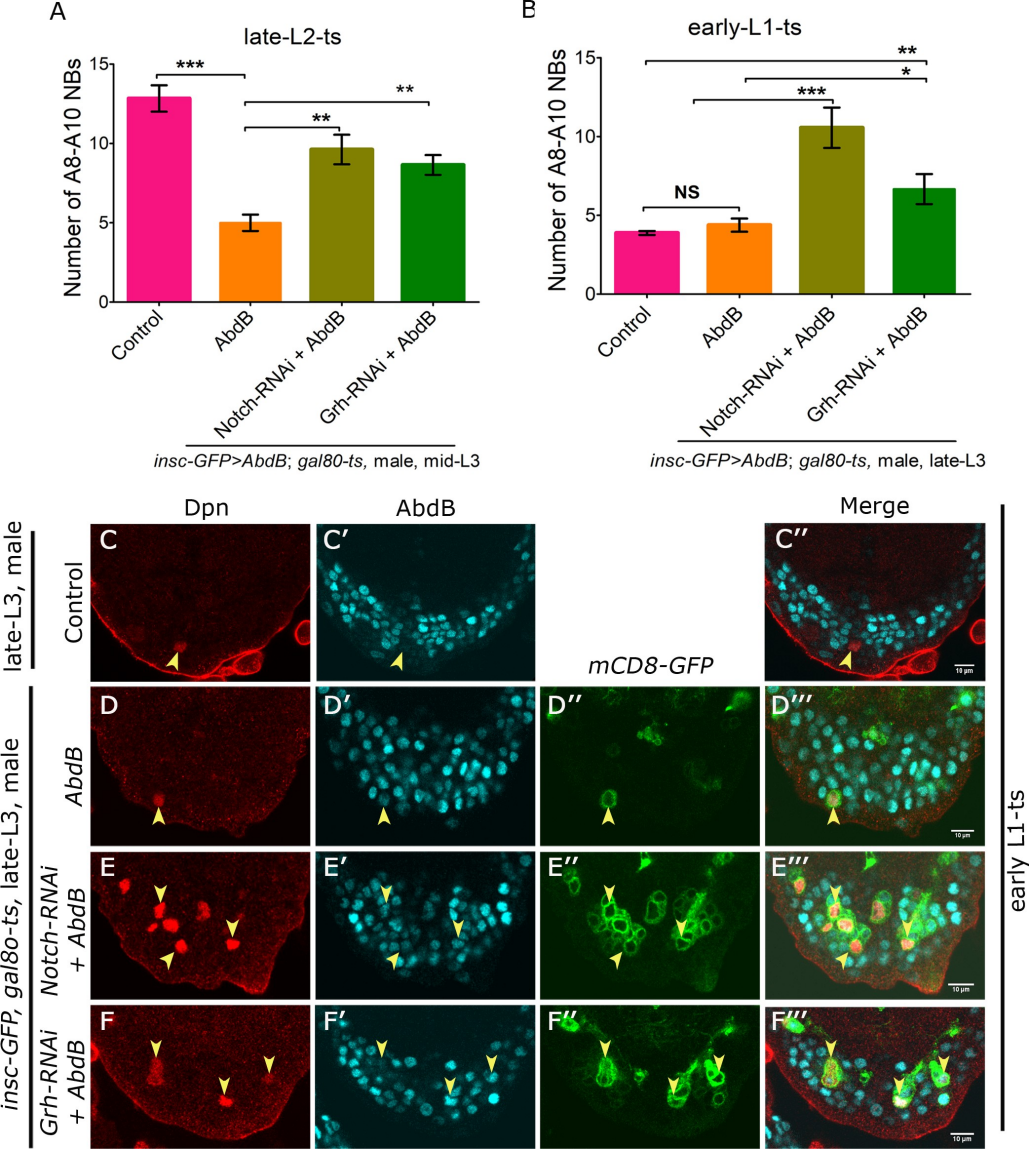
Notch and Grh give apoptotic competence to Dsx-negative NBs. (**A-B**) Graphs indicate that Dsx-negative NBs are capable of resisting Abd-B mediated apoptosis when Notch or Grh are knocked down by RNAi. (**A**) Shows surviving A8-A10 NBs for different genotypes (indicated on the x-axis) for late L2 to mid L3 stage temperature shift (TS, S1G Fig). (**B**) Shows surviving A8-A10 NBs for different genotypes (indicated on x-axis) for early L1 to late L3 stage temperature shift (TS, S1A Fig). (**C-D**) Male VNC at late L3 stage for control and Abd-B overexpression show 1 of the 4 Dsx-positive NBs surviving in A8-A10 segments. (**E-F**) Male VNCs at late L3 stage showing that inducing the knockdown for either Notch (**E**) or Grh (**F**) by RNAi (TS, S1A Fig) blocks the death of Dsx-negative NBs even with Abd-B overexpression. All the data shown is from male VNCs. Male VNC will have four Dsx-positive NBs which do not undergo apoptosis even on Abd-B overexpression (S8A and S8B Fig). Therefore, any NBs surviving in addition to these 4 NBs are Dsx-negative NBs. Yellow arrowheads indicate A8-A10 NBs. Scale bars are 10μm. Single confocal section is shown. Graph shows mean±s.d. Significance (*P*-value) is from two-tailed Student's unpaired *t*-test.

To test the role of Grh and Notch in determining apoptotic competence of Dsx-negative NBs, RNAi mediated knockdown (for Grh or Notch) and simultaneous overexpression of Abd-B were induced from late L2 stage and larvae were analysed in mid L3 stage (TS, S1G Fig). Owing to Abd-B’s capacity to cause sex-specific apoptosis in female by induction of Dsx^F^ [13] this experiment was carried out in male larvae. We found that control VNCs had close to 13 NBs in mid L3 stage (12.83+/- 2.04, n = 6 VNCs, N = 2, Fig 7A, bar-1), which included 4 Dsx-positive and remaining Dsx-negative NBs. Abd-B overexpression alone was sufficient to cause Dsx-negative NB apoptosis and reduce the total number of NBs to approximately 4 in mid L3 stage (5.2+/-1.30, n = 6 VNCs, N = 3) (Fig 7A, bar-2). But individual knockdown of either Notch (9.61+/-3.35, n = 13 VNCs, N = 3) or Grh (8.64+/-2.34, n = 14 VNCs, N = 3) in Abd-B overexpression background could resist this apoptosis (Fig 7A, bar-3 and 4). These numbers are relevant, considering that Dsx-negative NB apoptosis happens mainly in mid L3 stage. But to rule out the fact that the apoptotic resistance offered by knockdown of Grh and Notch was a result of limiting expression of Abd-B, the knockdown (for Grh or Notch) and Abd-B overexpression was induced for a longer duration (from early L1 to late L3 stage, TS, S1A Fig). Here as well we observed that both wild type control (3.80 +/- 0.35, n = 8 VNCs, N = 2, Fig 7B, bar-1 and Fig 7C–7C”) and Abd-B over expressed VNCs had only 4 Dsx-positive NBs surviving in late L3 stage (4.38+/-1.18, n = 8 VNCs, N = 3, Fig 7B, bar-2 and Fig 7D–7D”’), and all Dsx-negative NBs were dead. While, individual knockdown of either Notch (Fig 7E–7E”’ and Fig 7B, bar-3) or Grh (Fig 7F–7F”’ and Fig 7B, bar-4) could resist Abd-B induced apoptosis of A8-A10 NBs (10.55+/-3.84, n = 9 VNCs, N = 3 for Notch and 6.66+/-2.33, n = 7 VNCs, N = 3 for Grh, Fig 7B, bar-3 and 4).

We find that Grh expression and Notch activity increases in the temporal window prior to apoptosis in mid L3 stage and expression of Abd-B stays constant. Therefore, the capacity of Grh and Notch knockdown to resist the apoptosis induced by overexpression of Abd-B suggests that these two genes play an important role in determining the competence of Dsx-negative NBs to undergo apoptosis.

Grh is known to function as a tTF in embryonic NBs and has been shown to express in NBs prior to their quiescence at the end of embryogenesis [50]. In postembryonic stages, Grh is found to be expressed in abdominal NBs as early as L1 stage [5,7,12]. In terminal NBs we find that Grh expression is kept repressed till mid L2 stage (Fig 6A”) and is activated thereafter to bring about the apoptosis of these cells. Therefore, we tested if overexpression of Grh could advance this cell death from mid L3 stage to an earlier time point. We observed that overexpression of Grh (TS, S1H Fig) did not advance the death of Dsx-negative NBs to the early L3 stage and the number of A8-A10 NBs recovered in case of Grh overexpression (18.85+/-2.60, n = 6 VNCs, N = 2) was same as that of control male VNCs (18.57+/-2.99, n = 6 VNCs, N = 2, S9C and S9D Fig and S9F Fig). Similarly, we tested if we could advance the cell death of Dsx-negative NBs to the early L3 stage by increasing the Notch signalling. This was done by expression of NICD from early L1 stage in Dsx-negative NBs (TS, S1H Fig). In this case as well, we did not observe any precocious apoptosis of Dsx-negative NBs at early L3 stage. Instead we observed an increase in the number of A8-A10 NBs (39.66+/-5.85 n = 6 VNCs, N = 3) (S9C, S9E and S9F Fig). This observation corroborated an earlier reported role of NICD overexpression resulting in tumours in larval CNS [51]. These results indicated that while the temporal expression of both Grh and Notch are important in determining the apoptotic competence of Dsx-negative NBs, neither of them alone was a sole determinant of this competence.

## Discussion

Our results suggest that in case of Dsx-negative NB apoptosis, Abd-B and Su(H) bind on mutually exclusive DNA motifs found on apoptotic enhancer. By early L3 stage when Notch activity and Grh levels have sufficiently increased, apoptotic enhancer possibly assembles Abd-B-Grh and NICD-Su(H) complexes, which together activate *RHG* genes *grim* and *reaper* to cause NB apoptosis (S10 Fig).

### Role of NB proliferation, division mode and apoptosis in patterning of terminal VNC

In *Drosophila* the modification of abdominal and terminal segments starts in embryonic stages itself. This is mainly contributed by a gradient of NB proliferation as well as a switch in cell division mode (from Type I>0) along the AP axis (regulated by Hox genes). These molecular events together reduce the number of cells in these segments and lay the foundation of a wedge shaped CNS tapering towards the terminal region later in development [34, 35].

More specifically in case of terminal segments, Abd-B contributes to the wedge shape of the CNS during embryonic stages by following means. First, Abd-B prevents formation of certain lineages altogether in terminal segments of embryonic CNS by collaborating with TF Caudal [52]. Second, Abd-B reduces the proliferation of NBs in terminal segments compared to NBs in thoracic and abdominal segments [34, 35]. Third, Abd-B gives a more derived character to the NBs in the terminal region [52]. This results in a precocious switch in cell division mode (from Type I>0) for the terminal NBs as compared to abdominal and thoracic NBs [34, 35], thereby resulting in smaller lineages. These events are followed by Abd-B mediated apoptosis of majority of the NBs in terminal segments of embryonic CNS [4, 34], further limiting the number of cells in this region. Building on this molecular blueprint set up in embryonic stages, in post-embryonic (larval) stages Abd-B further reduces the neuronal numbers by causing the apoptosis of remaining NBs in A8-A10 segments [9, 13]. Abd-B does this by executing the death of both Dsx-negative NBs (this study) and Dsx-positive NBs (only in females) [9, 13], at different times using different molecular mechanisms (discussed below). Dsx-positive NBs in males however continue dividing and give rise to distinct population of serotonergic neurons involved in male mating behaviour [37–42]. However, it is not clear why a subset of NBs escapes embryonic apoptosis, undergoes quiescence and is eventually removed by another round of larval apoptosis soon after they exit quiescence.

### Divergent mechanisms of NB apoptosis in VNC

**Fig 8:**
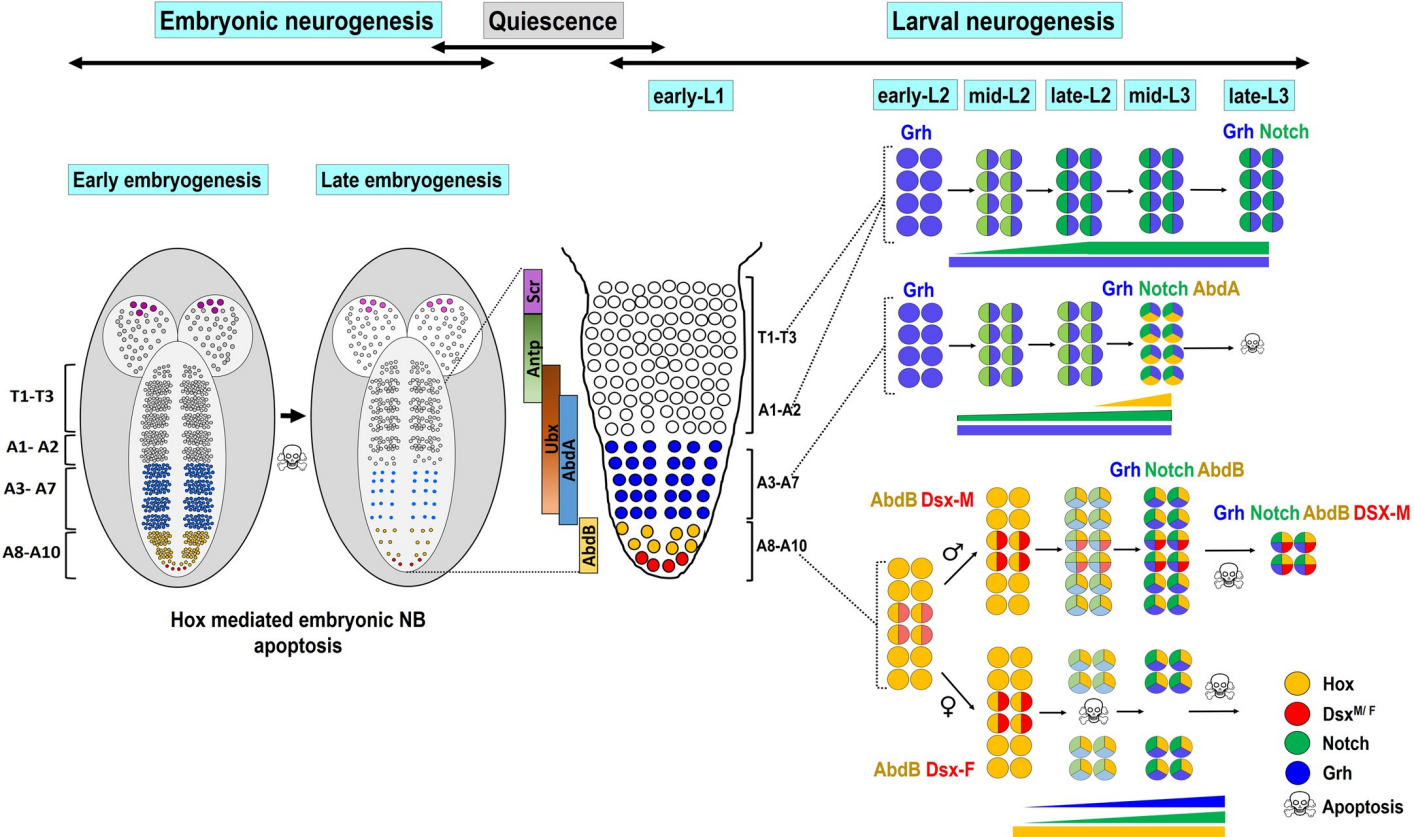
Increasing levels of Grh and Notch activity coupled with constant expression of Abd-B is required for Dsx-negative NB apoptosis. Schematic of embryonic CNS showing that NBs in T1-T3 and A1-A10 segments undergo Hox dependent apoptosis in late embryonic stages. The surviving NBs undergo quiescence and start dividing in early larval stages. Larval NBs in T1-A2 segments express Grh (shown in blue colour) and Notch (shown in green colour) but never express resident Hox gene (*antp*, *Ubx*, or *abd-A*) and hence continue dividing till late larval stages. The abdominal NBs (A3-A7 segments) express near constant levels of Grh (shown in blue colour) and Notch (shown in green colour). In these cells a mid L3 pulse of Abd-A (shown in yellow colour) results in their apoptosis by making them Abd-A^+^ Grh^+^ Notch^+^. In terminal segments Abd-B expresses in A8-A10 NBs (shown in yellow colour). Four of these NBs incrementally express Dsx (shown in deepening shade of red) and die in mid L2 stage by becoming Abd-B^+^ DsxF^+^ in females VNC, while their male counterparts (which become Abd-B^+^ DsxM^+^) continue dividing till pupal stages. The Dsx-negative NBs on other hand show an increase in Notch activity and Grh expression (indicated by deepening shades of green and blue colour), coupled with constant levels of Abd-B (shown in yellow colour) and undergo apoptosis (by becoming Abd-B^+^ Grh^+^ Notch^+^) at mid L3 stage.

Hox mediated NB apoptosis plays an important role in generating the correct number of cells across the AP axis of developing CNS [4, 5, 7–13]. In abdominal and gnathal segments, apoptosis occurs in response to NBs switching from TF code of Hox^-^ Grh^+^ to Hox^+^ Grh^+^ in mid L3 stage [7, 12] (Fig 8). However, we find that Abd-B expressing terminal region is more complex and has different dedicated mechanisms for apoptosis of Dsx-positive and Dsx-negative NBs, which die at different times during development. Dsx-positive NBs undergo sex-specific apoptosis using Abd-B and Dsx^F^ in females at mid L2 stage (when Grh and Notch are not expressed in these cells). This female specific cell death relies on formation of a cooperative complex of Abd-B and Dsx^F^ on specific motifs of the apoptotic enhancer [13]. Whereas Dsx-negative NBs undergo apoptosis much later in mid L3 stage (same time as A3-A7 NBs) through a mechanism using Hox, Grh and Notch signalling instead of Hox-Dsx (S10 Fig). Expectedly, motifs used by abdominal NBs for maintenance of enhancer activity are also used in Dsx-negative NBs. Interestingly, we observed that mutagenesis of Su(H) binding sites on the enhancer did not abrogate the lacZ expression in 37% of the NBs. This indicated that there are additional Su(H) binding sites in the enhancer that are used for maintaining its activity *in vivo*. These unidentified sites are most likely different from the consensus Su(H) binding sequence (of RTGRGAR [53]) used in our previous analysis [12]. However, this does not rule out the role of mutagenized Su(H) sites in the enhancer activity maintenance, as we observe a significant decrease in lacZ expression as well as a reduction in percentage of NBs expressing *Su(H)*^*mutant*^*-lacZ* (Fig 5F and 5G).

The collaboration of Grh and Notch with Hox in the apoptosis of NBs is already reported in larval abdominal (A3-A7) segments [12]. In these cells, increasing levels of Hox factor Abd-A function as a trigger for apoptosis [5], while Grh levels and Notch activity mostly remains constant (S7 Fig; summarized in Fig 8). In these segments Grh is expressed in NBs from embryonic stage 15 onwards [4, 50, 54] and plays a dual role in larval NB apoptosis, by sustaining the Abd-A expression in abdominal NBs, as well as simultaneously facilitating their death [7].

Contrary to this, in case of terminal segments, we find that there is no cross regulation between Abd-B, Grh and Notch in NB apoptosis. We also observe that in terminal segments, Grh is reactivated in early larval stages (Fig 6B”). This increase in Grh expression is preceded by an increase in Notch activity. These two events coupled with constant levels of terminal Hox gene Abd-B are utilized to cause Dsx-negative NB apoptosis (summarized in Fig 8). This underlines notable commonalities and differences between the two mechanisms used in abdominal and terminal regions. Commonalities being apoptotic enhancer, timing of death and molecular players (Notch, Grh and Hox) involved. While the differences being mechanism of death initiation (by distinct temporal activation of molecular players), Hox genes involved in abdominal and terminal segments, and the fact that terminal Hox Abd-B does not utilize its canonical cofactor Exd in this apoptosis.

While the immediate significance of the apoptosis of Dsx-negative NBs is not clear, but the neural circuitry in this region has been shown to play a role in male mating behaviour as well as female receptivity towards mating [41, 42]. This suggests that additional neurons, surviving in case of the death defects, are likely to interfere with adult copulation, similar to what has been reported for male flies that are mutant for *RHG* genes. These mutant males were unable to mount and copulate properly [33]. Furthermore, what is intriguing is why would cells in abdominal and terminal regions use common players but employ different molecular mechanisms to initiate *RHG* gene expression and eventually cause apoptosis. One possibility is that Abd-B may have a stronger binding on the enhancer compared to Abd-A. Therefore, while the cells in abdominal segments could gradually activate *RHG* genes in a calibrated manner in response to increasing levels of Abd-A, such molecular calibration may not be possible with Abd-B. Thus, in terminal segments, temporal increase in levels of Grh and Notch signalling may have been evolved as an alternative strategy, which can be used for a similar molecular calibration to integrate different inputs on the apoptotic enhancer. Alternatively, the difference in mechanism could be because Abd-B is required at an earlier time point for induction of Dsx expression in a sub-population of A8-A10 NBs, which then results in sex-specific death of Dsx-positive NBs in females. The abdominal NBs on other hand do not have any sex-specific alternative fates.

Interestingly, despite Dsx-positive NBs in males expressing Abd-B and showing increasing Notch activity and Grh expression, these cells do not undergo apoptosis. This is probably due to expression of Dsx^M^ isoform in these cells, which, amongst other roles have a dominant anti-apoptotic function [9, 13], and therefore leads these cells to a different fate altogether. This further underlines the theme of overlapping molecular players resulting in different cell fates based on their interacting partners.

Most of the NBs undergo cell cycle exit during early pupal stages, except Murshoom body NBs (MBNBs) [55, 56], which are eliminated by apoptosis and autophagy in mutually exclusive manner [57, 58]. It will be interesting to test whether Grh expression and Notch activity are maintained in MBNBs till late pupal stages. Moreover, in absence of any visible Hox gene expression in these cells, how other spatial TFs could play a role in coordinating their cell death remains an open question.

## Materials and methods

### Fly stocks

Fly stocks used: *M22/TM6Tb* [12]; *FRT-hth*^*P2*^ (R.Mann [59]), *FRT-exd*^*1*^ (BDSC 3293); *grim*^*A6C*^*-rpr*^*17*^*/TM6Tb* [13]; *MM3/TM6Tb* and *grimA6C/TM6Tb* [36]; *FRT-Abd-B-M*^*1*^ [60]; *Df(2R)Pcl7B* [7]; *grh*^*370*^/*SM6 CyO*, *E(spl)mγ-GFP* [45]; *hsflp*, *FRT19A*, *tub-GAL80*; *tub-GAL4*, *UASmCD8*::*GFP*/*SM6 CyO-GFP* (H. Reichert [5]); *repo-GAL4*::*GFP* (Chr. III, BDSC 7415); *repo-gal4* (Chr. II, Bradley Jones [44]); *grh[NB-D4]-gal4/TM6B* (S. Bray) [45]; *elavGAL4* (BDSC 8765); *worniu-GAL4* (Chris Doe); *UAS-dcr2; inscGAL4 UASmCD8-GFP and UAS-dcr2; inscGAL4 UASmCD8-GFP; tub-GAL80*^*ts*^ (J. A. Knoblich [61]); *Canton-S* (BDSC 64349); *UAS-NICD* (BDSC 52008); *UAS-p35* (DGRC 108019); *Notch-GFP* (BDSC 81271); *UAS-Abd-B* (DGRC 106120); *UAS-grh-RNAi*, *UAS-Abd-B* (this study); *elav[C155]-GAL4*, *UAS-mCD8-GFP*, *hsflp1*, *w* (BDSC, 5146)*; yw; tub-GAL80-LL9 FRT80B* (BDSC, 5191); *yw*; FRT82B *tub-GAL80-LL3* (BDSC 5135); *Notch-RNAi* (BDSC 28981), *Dl-RNAi* (BDSC 34322); *Abd-B-RNAi* (VDRC 12024), *grh-RNAi* (VDRC 101428) *abd-A*-*RNAi* (VDRC 106155), *exd-RNAi* (VDRC 7802) and *hth-RNAi* (NIG-17117-R4 and R2). *F3B3-lacZ*; *717-lacZ*, *Grh*^*mutant*^*-lacZ*; *Su(H)*^*mutant*^*-lacZ*; *Hox-Exd-Grh*^*mutant*^*-lacZ (AT-Grh*^*mutant*^*-LacZ)* [12]; *Hox-Exd*^*mutant*^*-lacZ (AT*^*mutant*^*-LacZ)* (this study). All transgenic lines were generated by site specific insertion [62] of the mutagenized *enhancer-lacZ* fusion construct at attP40-25C6. *UAS-GrhO* was generated by classical P-element based transgenesis.

### CRISPR-Cas9 deletion of 717bp enhancer

The deletion of the 717 bp was performed using double gRNA/Cas9-based strategy as described in Kondo et al 2013 [63]. The following guide RNA sequences were chosen:

gRNA-1 (for the left breakpoint): GGTACAGCCTCCAAAAGGGC
gRNA-2 (for the right breakpoint): GACCAAACGAAAGGGACTTA

Both the sequences were cloned into a double gRNA vector where each of the guides is expressed under its own U6 promoter. The constructs were then integrated into the attP40 landing site on the second chromosome. The double gRNA containing males were then crossed to females of nos-cas9 and the resultant offspring containing both the gRNA and the cas9 were obtained. These were then crossed to third chromosome balancer flies and subsequently 20 independent stocks were established. The homozygous flies from each stock were then tested for the presence or absence of deletion by PCR. Two independent stocks containing the desired deletion were obtained and the breakpoints were mapped by PCR. The following primer pairs were used to screen for the deletion:

*Amplicon A*: *TTGCCCAACACGGATCGATGAG and CCTCAACGTTCACTCTTGTTTCC*
*Amplicon B*: *TTGCCCAACACGGATCGATGAG and GGAGTACAAATATCAGGCACTG*

### Fly husbandry

All the fly stocks and crosses were maintained at 25°C unless otherwise mentioned. For fly crosses, 4-hour window egg collections were done and the larvae were reared at 25°C or 29°C (for RNAi knockdown/ overexpression experiments). All the larvae were dissected at the desired larval stages. Details of the genotypes analysed in different figure panels are given in S1 Data.

### Immunohistochemistry and image acquisition

Larvae of the desired age, genotype and sex were dissected as described earlier [13] with the following variations; fixation was done for 30mins at room temperature (RT) and immunostaining with primary antibodies overnight at 4°C. The following primary antibodies were used: rabbit anti-dcp-1 (1:10, CST 9578), rabbit anti-Caspase-3 (1:10, CST, 5A1E); rabbit anti-Dpn, 1:5000; rat anti-Dpn, 1:2000; mouse anti-Grh, 1:2000; rabbit anti-Grh, 1:2000 [12], mouse Abd-B (1:50, 1A2E9, DSHB), mouse anti-NICD (1:100, C17.9C6, DHSB), mouse anti-Repo (1:100, 8D12, DHSB), mouse anti-Dl (1:10, C594.9B, DHSB), rat anti-Dpn (this study), rat anti-Dsx (1:3000) [13], chicken anti-GFP (1:2000, ab13970, Abcam) and chicken anti-β-gal (1:2000, ab9361, Abcam). Secondary antibodies conjugated to Alexa fluorophores from Molecular Probes were used: AlexaFluor405 (1:250); AlexaFluor488 (1:500); AlexaFluor555 (1:1000); and AlexaFluor647 (1:250). All the brain samples were mounted in 70% glycerol and the fluorescent images were acquired using ZEISS LSM 700 inverted confocal microscope and analysed using ZEN 2012 software. The NBs were counted manually by cell counter (plugin in ImageJ) scanning the entire scans in the region of interest, while sufficiently taking care that no cell is counted twice. In all the images yellow and white arrowheads represent NBs while arrows represent GMCs. Scale bars (10 micron) are shown in merge figures. All the quantifications were done in Microsoft excel, while graph plotting and statistical data analysis (paired and unpaired student t-test, one way ANNOVA test) was performed using GraphPad Prism software.

### Estimation of mean fluorescence intensity

All mean fluorescence intensities were estimated in ImageJ software by drawing a circle around Dpn positive NB and then measuring the intensity in all other given channels. The calculated intensities were subtracted by background mean intensity from neuropil of the respective channel. Percentage of NB expressing a particular epitope was calculated by manually counting the lacZ, Grh or E(spl)mγ-GFP expression status of the NBs.

### Temperature shift experiments

Fly crosses were set up between virgin females of genotype, *UAS-dcr2; inscGAL4-UASmCD8-GFP; tub-GAL80*^*ts*^ and males of required genotype- *UAS-p35; UAS-Abd-B-RNAi; UAS-Grh-RNAi; UAS-Notch-RNAi; UAS-Dl-RNAi; UAS-Exd-RNAi; UAS-Hth-RNAi; UAS-Grh; UAS-Abd-B; UAS-NICD; F3B3-LacZ*, *UAS-Grh-RNAi; F3B3-lacZ*, *UAS-Notch-RNAi; F3B3-lacZ*, *UAS-Abd-B-RNAi; UAS-Abd-B*, *UAS-Grh-RNAi and UAS-Abd-B*, *Notch-RNAi* with suitable controls. 4 hrs egg collections were done and the larvae were reared at 18°C till the desired stage and were then shifted to 29°C. All fly crosses using *repo-gal4*, *wor-gal4*, *elav-gal4* and *grh-gal4* were set up and reared at 25°C for 24 hours before shifting to 29°C till the desired time for dissection. The larvae were sexed, separated at specific times and subsequently dissected as per the requirement of each individual experiment. The schematic of temperature-shift (TS) protocol for each experiment is mentioned in text and summarized in S1 Fig.

L1 and L2 stages were divided into three 8hrs interval which defined early, mid and late stage for these two stages. L3 stage was divided into three 16 hr interval to define early, mid and late L3 stage. In all figures sex of the dissected larvae is mentioned on each figure panels, except for the cases where a mixed population was analysed.

### Mosaic analysis of cell repressible marker (MARCM)

MARCM experiments were performed as per previously described protocol [64]. The following genotypes were crossed in order to generate the desired clones:

*FRT-Abd-B*^*M1*^ MARCM clones: virgin females of the genotype *elav[C155]-GAL4*, *UASmCD8*::*GFP*, *hsflp1*, *w; FRT82B tub-GAL80-LL3* were crossed to males of *FRT-Abd-B*^*M1*^ /*TM6Tb*.
*FRT-exd*^*1*^ MARCM clones: virgin females of the genotype *FRT-exd*^*1*^/*FM7* were crossed to males of *hsflp*, *FRT19A*, *tubGAL80; tub-GAL4*, *UASmCD8GFP/SM6 CyO-GFP*.
*FRT-hth*^*P2*^ MARCM clones: virgin females of genotype *elav[C155]-GAL4*, *UASmCD8*::*GFP*, *hsflp1*, *w; FRT82B tub-GAL80-LL3* were crossed to males of *FRT-hth*^*P2*^*/TM6Tb*.

All the eggs collected for a duration of 4 hours at 25°C were subjected to periodic heat-shock of 1 hour at 37°C after every 12 hour starting from the time of larval hatching to mid third instar larval stage. The dissections were performed at the late third instar larval stage.

### Electrophoretic Mobility Shift Assay (EMSA)

EMSAs were performed as described previously [12]. For EMSA binding studies following protein constructs were used: N-terminal-GST-tagged-Abd-B-N (aa residues 313 to 493) [13] and 6XHis tagged Grh (551–1333 aa) (in S3 Fig). All the binding reactions were setup in a 20 μl volume and incubated at room temperature for 40 minutes.

### GST pull-down experiments

The following constructs were used for the affinity pulldown assay: N-terminal-GST-tagged fusion proteins were used for Abd-B-N (aa residues 313 to 493) and His tagged Grh (551–1333 aa). Bacterial cultures of GST tagged Abd-B-N and His tagged Grh were induced at OD600 with 0.5 mM IPTG for 2 h and 3 h at 18°C. The affinity pulldown experiments were performed as described in a previous report [13].

## Supporting information

S1 Fig Approximate timing for the various temperature shift experiments. (A-H) The temperature shift (TS) protocols used in different experiments are shown in different panels. The downward facing arrow indicates the time of dissection of the larvae. L1 and L2 stages were divided into three 8hr intervals which defined early, mid and late stages. L3 stage was divided into three 16 hr intervals to define early, mid and late L3 stages.
(TIF)

S2 Fig A8-A10 NBs express apoptotic markers. (A-C) Show the expression of apoptotic markers Dcp-1 and Casp-3 in A8-A10 NBs of female VNCs in mid L2 (A) and mid L3 stage (B-C). Dsx-positive NBs in female VNC die at mid L2 stage, therefore Dcp-1/Casp3 positive NBs in CNS at mid L3 stage are Dsx-negative NBs. (D-E) Show that expression of p35 (D) and knockdown of Abd-B (E) results in a block of Dsx-negative NB apoptosis in late L3 stage female VNCs. (F) Shows that knockdown of Abd-A by RNAi from early L1 stage (TS, S1A Fig) results in a block of NB apoptosis in A3-A7 segments in female VNC at late L3 stage. (G) Graph showing the number of surviving NBs in A3-A7 segments of late L3 female VNCs when *abd-A-RNAi* is induced from early L1 stage. Yellow arrowheads indicate Dsx-negative NBs in panels “A-E”. Scale bars are 10μm. All images are single confocal sections except for panel “F” which is a partial z-project of confocal stacks. Graph shows mean±s.d. Significance (*P*-value) is from two-tailed Student's unpaired *t*-test.
(TIF)

S3 Fig Expression of Notch ligand Delta across different stages in A8-A10 NBs. (A-E) Show Notch-GFP marked NBs and associated lineages expressing Notch ligand Delta in female VNCs at early L2 (A), late L2 (B), early L3 (C), mid L3 (D) and late L3 stages (E). (F) Shows Delta staining in NBs and associated lineages in female VNC at late L3 stage wherein expression of p35 blocks the death of A8-A10 NBs (TS, S1A Fig). (G) Shows lack of Delta staining in NBs and associated lineage when Delta is knocked down by RNAi (TS, S1A Fig), indicating that Delta staining in specific. Yellow arrowheads indicate A8-A10 NBs. Scale bars are 10μm. All images are single confocal sections.
(TIF)

S4 Fig Expression of *grh-GAL4* and *717-lacZ* in NBs across different stages. (A-D) Show that *grh-GAL4* driven expression of UAS-nls-GFP closely correlate with Grh protein expression in abdominal and terminal NBs in early L2 (A), late L2 (B), early L3 (C) and mid L3 stages (D). (E-G) Shows that compared to wild type, *grim*^*A6C*^*-reaper*^*17*^ homozygous double mutants and *MM3/M22* transheterozygotes show a block of Dsx-negative NB apoptosis in late L3 female VNCs. (H-J) Shows the expression of *717-lacZ* in A8-A10 NBs in female VNCs in mid L2 (H), early L3 (I) and mid L3 (J) stages. (K) Graph showing the quantitation of lacZ intensity in A8-A10 NBs across different stages. Panels “E-G” are single confocal sections; rest all panels are partial z-projects. Yellow arrowheads indicate A8-A10 NBs. White arrowheads in panels “A-D” show abdominal NBs and in panels “F and G” indicate Dsx-positive NBs. Scale bars are 10μm. Graph shows mean±s.d. Significance (*P*-value) is from two-tailed Student's unpaired *t*-test.
(TIF)

S5 Fig Grh and Abd-B binding on 717bp. (A-I) EMSA autoradiogram for Abd-B, Grh and Abd-B-Grh binding on oligonucleotides for maintenance motifs-25 (A), 27 (E), 28 (D), 30 (B), 31 (C), 32 (G), 33 (F) and 34 (H) of 717bp enhancer are shown. Wild type and Hox mutant oligonucleotide sequences used are shown at the bottom of each gel. Hox and Grh binding sequences are colour coded in red and blue respectively. Mutations are shown in small case. Only motif-33 (F) shows Abd-B-Grh-DNA complex formation. (I) Shows a schematic of 717bp enhancer showing the relative position of various binding motifs. Proteins added to a specific lane are shown at the top of each lane. Lane with free probes are indicated by downward facing black arrows. EMSA indicate that Abd-A binding sites are also capable of binding Abd-B. Blue rectangles indicate a constant concentration of 200ng for Grh protein. An increasing concentration of 50ng and 100ng of Hox are indicated by red right triangles. Red arrow heads on the gels indicate Hox-DNA complex; while blue arrow heads indicate the Grh-DNA complex. Black arrowhead in panel “F” indicates Grh-Abd-B-DNA complex.
(TIF)

S6 Fig Su(H) binds on 717bp enhancer and interacts with Abd-B and Grh *in vitro*. (A) Shows schematic of 1Kb *F3B3-lacZ*, *717-lacZ* and its mutant versions *Grh*^*mutant*^*-lacZ*, *AT*^*mutant*^*-lacZ*, *AT-Grh*^*mutant*^*-lacZ* and *Su(H)*^*mutant*^*-lacZ* (used in Fig 5 of the main text). Motifs with Grh binding sites are shown as green rectangles (if they have a single Grh binding site) and as green squares (if they have two Grh binding sites). Su(H) binding sites are indicated as blue squares and asterisk indicates the Su(H) binding site for which EMSA is shown in (B). Black crosses indicate mutagenesis of AT rich and Su(H) sites. “G” in white font within green boxes indicate mutation of just Grh binding sites within these motifs. (B) Shows EMSA autoradiogram for Su(H) binding site indicated by asterisks on 717bp enhancer. Out of seven sites shown on the schematic only one site (indicated by asterisk) showed an *in vitro* binding by Su(H) (indicated by black arrowhead). (C) EMSA autoradiogram showing that Su(H) does not bind on motif-30 which has Hox-Exd-Grh binding sites, lane-1 and 4 show free probes. Right triangle on top of EMSA indicates an increasing concentration of 150 and 300 ng of Su(H) protein. Sequence of oligonucleotides is shown at the bottom of EMSA. (D) Western blot showing in-vitro pulldown assay for bacterially expressed GST-tagged Abd-B (but not GST alone) is able to pull down His-tagged Su(H) (lane-8 vs 9). (E) Western blot showing in-vitro pulldown assay for bacterially expressed GST-tagged Su(H) (but not GST alone) is able to pull down His-tagged Grh (lane-11 vs 12). Coomassie Blue depicts almost equal loading of the GST-tagged protein samples in D and E.
(TIF)

S7 Fig Expression of Grh and E(spl)-mγ-GFP stays relatively unchanged in abdominal NBs from early L2 to mid L3 stage of development. (A-D) Show expression of Grh and E(spl)mγ-GFP across early L2 (A), late L2 (B), early L3 (C) and mid L3 stages (D) in abdominal and terminal NBs of female VNCs. (E-G) Show graphs depicting quantitation of E(spl)mγ-GFP (E), Grh (F), and control Dpn staining (G) intensities across different stages. Graphs indicate that expression of Grh and E(spl)mγ-GFP is mostly constant across different stages in abdominal NBs. Slight but significant difference in E(spl)mγ-GFP expression is seen from early L3 to mid L3 stage. Partial z-projects are shown for A-D to show both abdominal and terminal NBs (indicated by white and yellow arrowheads respectively). Scale bars are 10μm. Graph shows mean±s.d. Significance (*P*-value) is from two-tailed Student's paired *t*-test.
(TIF)

S8 Fig Abd-B, Grh and Notch do not show any cross-regulation in A8-A10 NBs. (A-B, D-E) Show that compared to A8-A10 NBs in control VNCs, knockdown of Grh (A-B) and Abd-B (D-E) does not affect E(spl)mγ-GFP expression in mid L3 stage VNCs. (C, F) Graphs showing quantitation of E(spl)mγ-GFP fluorescence in A8-A10 NBs of control VNCs versus Grh knockdown (C) and Abd-B knockdown (F). (G-I) Show that compared to p35 expressing A8-A10 NBs, knockdown for Grh (H) or Notch (I) (TS, S1A Fig) does not affect expression of Abd-B, or Abd-B and Grh respectively. (J) Shows the graph comparing the levels of Abd-B in A8-A10 NBs at late L3 stage in p35 expressing VNCs versus Notch or Grh knockdown (induced from early L1 stage, TS, S1A Fig). (K) Shows the graph comparing the levels of Grh in A8-A10 NBs at late L3 stage in p35 expressing VNCs versus VNCs with Notch knockdown (induced from early L1 stage, TS, S1A Fig). (L-M) Show that compared to control VNCs knockdown of Abd-B from early L1 stage does not affect the expression of Grh in A8-A10 NBs at mid L3 stage (TS, S1G Fig). (N-O) Show the graphs comparing the levels of Grh (N) and Abd-B (O) in A8-A10 NBs at mid L3 stage in control VNCs versus Abd-B knockdown (N) and for control VNCs versus Grh knockdown (O) (TS, S1F Fig). These results indicate that unlike in A3-A7 segments Abd-B, Grh and Notch does not show any cross regulation in A8-A10 segments. Both male and female VNCs were used in these experiments. Yellow arrowheads indicate A8-A10 NBs. Scale bars are 10μm. All images are single confocal sections. Graph shows mean±s.d. Significance (*P*-value) is from two-tailed Student's unpaired *t*-test.
(TIF)

S9 Fig Overexpression of Grh and NICD does not advance the time of apoptosis for Dsx-negative NBs from mid L3 stage of development. (A-B) Show that compared to control male VNC overexpressing GFP (A), overexpression of Abd-B in Dsx-positive NBs does not result in their apoptosis, indicating that Dsx-positive NBs in males are refractory to apoptosis induced by Abd-B. (C-E) Show that compared to controls (C) overexpression of Grh (D) or NICD (E) from early L1 stage (TS, S1A Fig) does not advance Dsx-negative NB apoptosis to an earlier time point of early L3 stage. (F) Shows a graph comparing the number of surviving A8-A10 NBs at early L3 stage for Grh and NICD overexpression compared to controls. Male VNCs are shown. Yellow arrowheads indicate A8-A10 NBs. Scale bars are 10μm. All images are single confocal sections. Graph shows mean±s.d. Significance (*P*-value) is from two-tailed Student's unpaired *t*-test.
(TIF)

S10 Fig Schematic comparing the molecular basis of apoptosis of Dsx-positive versus Dsx-negative NBs. Dsx-positive NBs die at mid L2 stage, wherein Abd-B (shown in purple) and Dsx^F^ (shown in red) cooperate to activate apoptotic enhancer to cause female specific apoptosis. This happens on a specific set of Abd-B-Dsx binding motifs (shown by green boxes) on the enhancer (shown by black line). While Dsx-negative NBs undergo apoptosis in mid L3 stage relying on presence of Abd-B, Grh (shown by dark green filled circle) and Notch (shown in dark blue triangle) using a completely different set of binding motifs (shown as light blue boxes). These motifs are required for maintenance of the apoptotic enhancer in Dsx-negative NBs. Abdominal NBs in A3-A7 segments also rely on the same motifs (shown as light blue boxes) for maintaining the activity of the enhancer in abdominal NBs.
(TIF)

S1 Data Supplementary data for additional experiments and details of the genotypes analysed in different figure panels. (DOCX)
