# Supplementary figures and images for: Sequential activation of Notch and Grainyhead gives apoptotic competence to Abdominal-B expressing larval neuroblasts in Drosophila Central nervous system
Source: PLoS Genet. 2020 Aug 31;16(8):e1008976. doi: 10.1371/journal.pgen.1008976 (PMC7485976; doi:10.1371/journal.pgen.1008976)

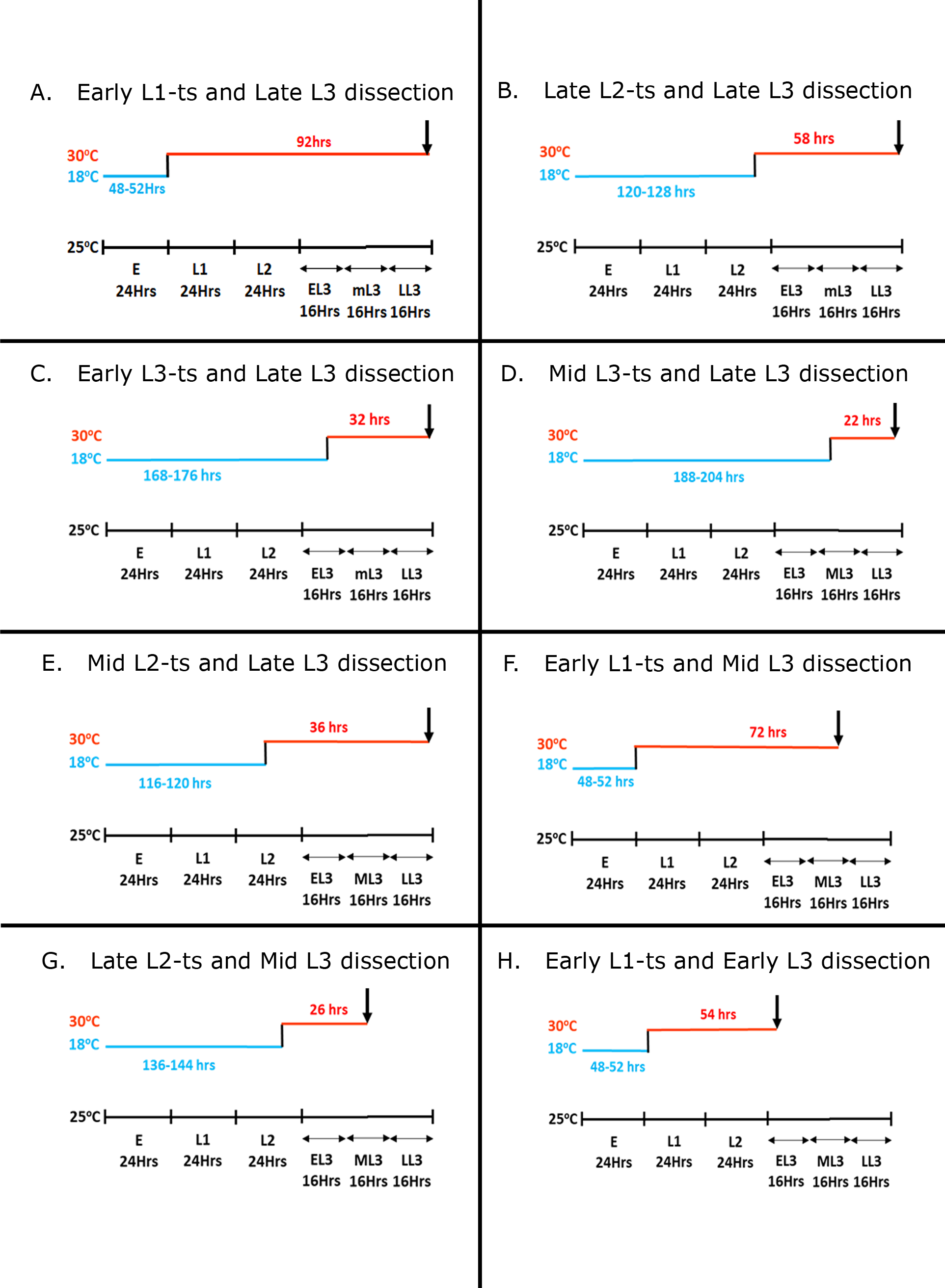

Supplement: S1 Fig — (A-H) The temperature shift (TS) protocols used in different experiments are shown in different panels. The downward facing arrow indicates the time of dissection of the larvae. L1 and L2 stages were divided into three 8hr intervals which defined early, mid and late stages. L3 stage was divided into three 16 hr intervals to define early, mid and late L3 stages. (TIF) [file pgen.1008976.s001.tif]

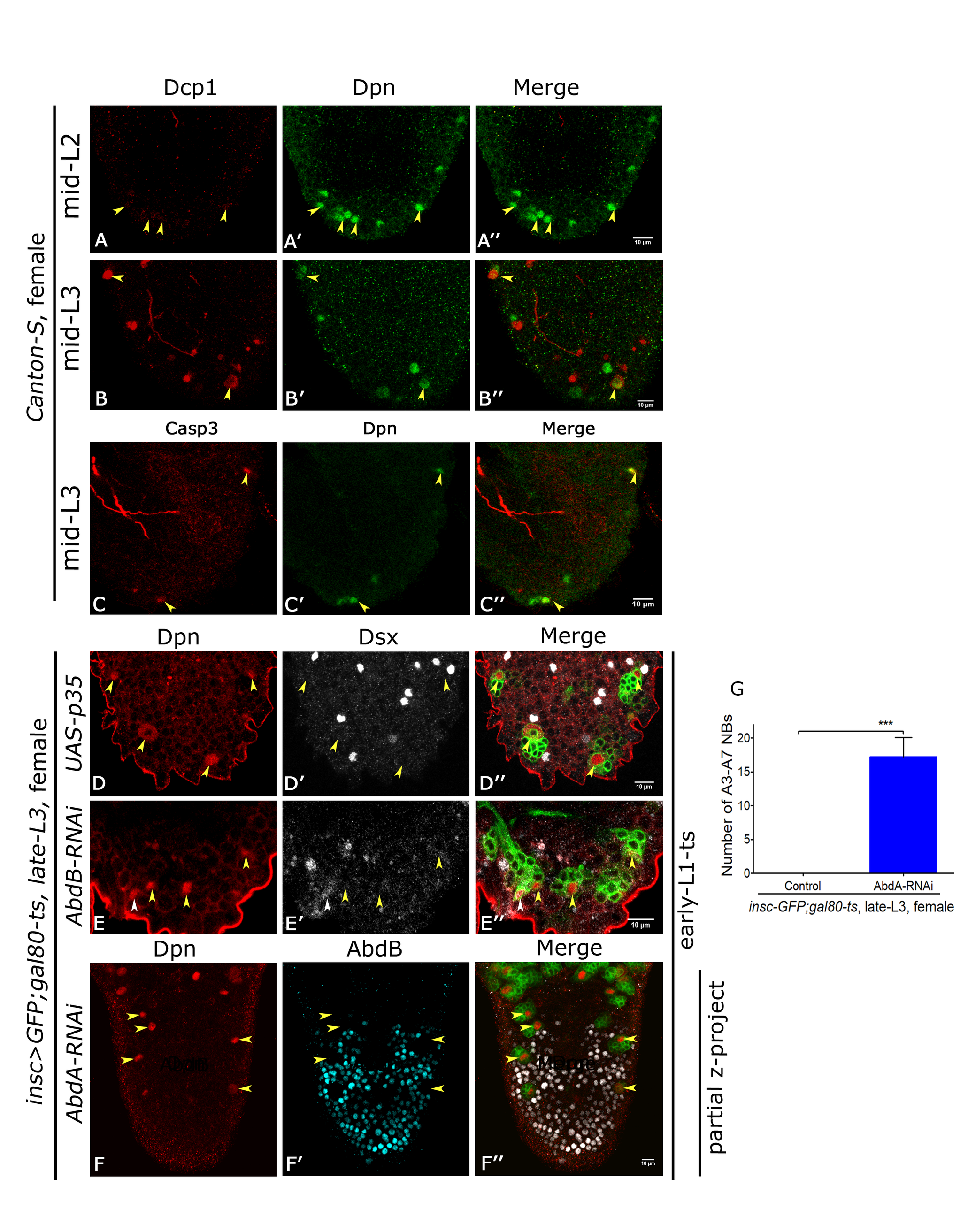

Supplement: S2 Fig — (A-C) Show the expression of apoptotic markers Dcp-1 and Casp-3 in A8-A10 NBs of female VNCs in mid L2 (A) and mid L3 stage (B-C). Dsx-positive NBs in female VNC die at mid L2 stage, therefore Dcp-1/Casp3 positive NBs in CNS at mid L3 stage are Dsx-negative NBs. (D-E) Show that expression of p35 (D) and knockdown of Abd-B (E) results in a block of Dsx-negative NB apoptosis in late L3 stage female VNCs. (F) Shows that knockdown of Abd-A by RNAi from early L1 stage (TS, S1A Fig) results in a block of NB apoptosis in A3-A7 segments in female VNC at late L3 stage. (G) Graph showing the number of surviving NBs in A3-A7 segments of late L3 female VNCs when abd-A-RNAi is induced from early L1 stage. Yellow arrowheads indicate Dsx-negative NBs in panels “A-E”. Scale bars are 10μm. All images are single confocal sections except for panel “F” which is a partial z-project of confocal stacks. Graph shows mean±s.d. Significance (P-value) is from two-tailed Student's unpaired t-test. (TIF) [file pgen.1008976.s002.tif]

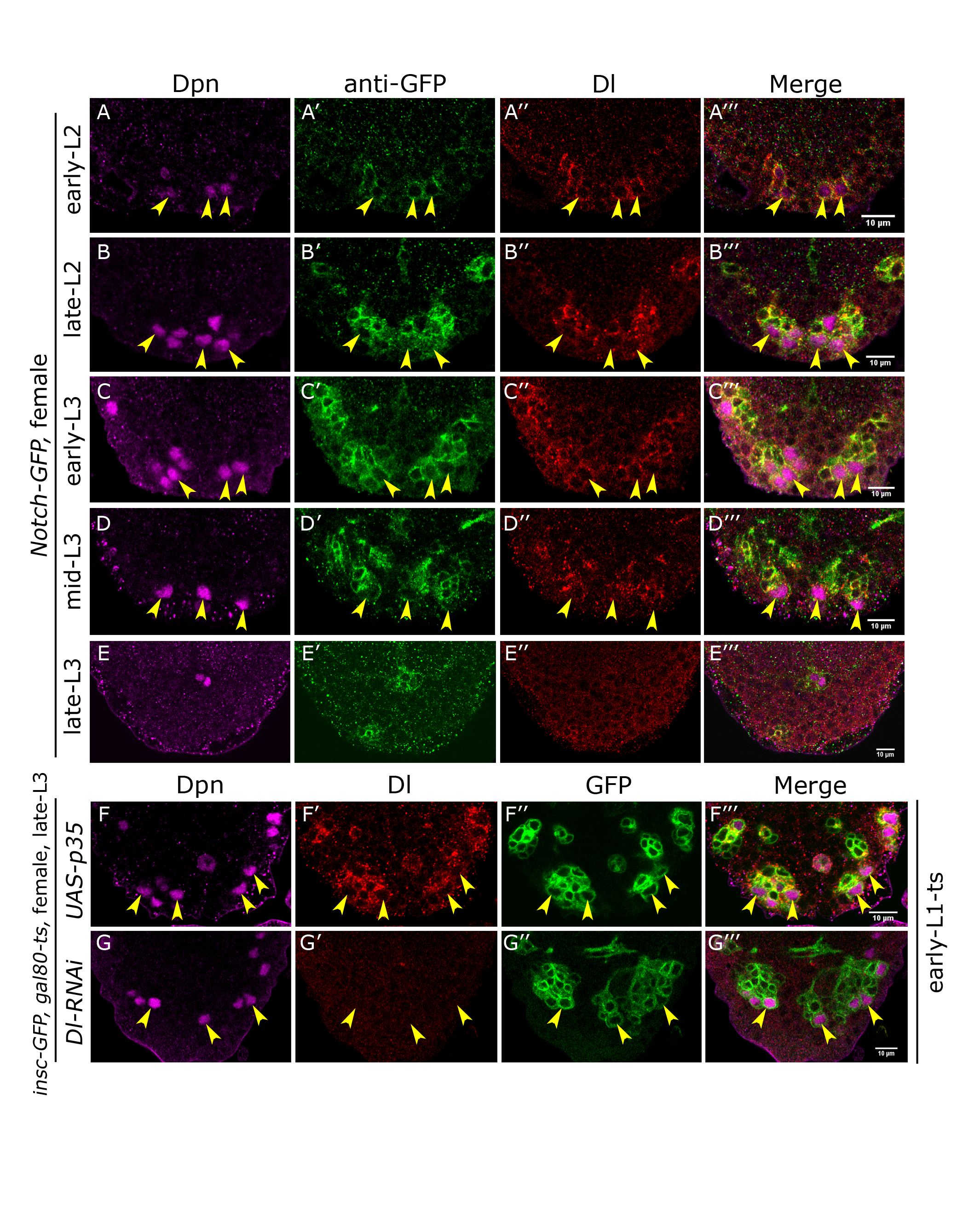

Supplement: S3 Fig — (A-E) Show Notch-GFP marked NBs and associated lineages expressing Notch ligand Delta in female VNCs at early L2 (A), late L2 (B), early L3 (C), mid L3 (D) and late L3 stages (E). (F) Shows Delta staining in NBs and associated lineages in female VNC at late L3 stage wherein expression of p35 blocks the death of A8-A10 NBs (TS, S1A Fig). (G) Shows lack of Delta staining in NBs and associated lineage when Delta is knocked down by RNAi (TS, S1A Fig), indicating that Delta staining in specific. Yellow arrowheads indicate A8-A10 NBs. Scale bars are 10μm. All images are single confocal sections. (TIF) [file pgen.1008976.s003.tif]

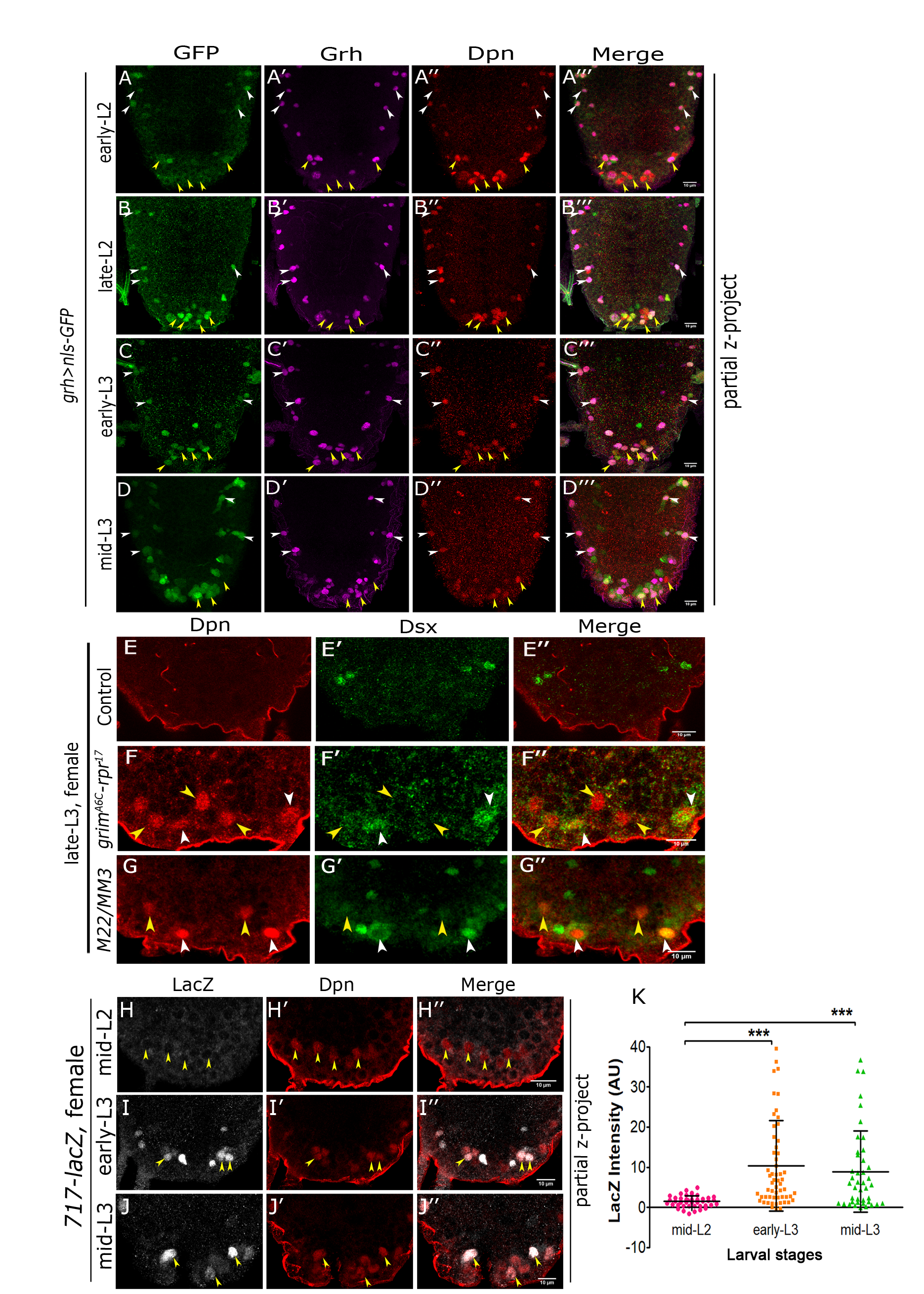

Supplement: S4 Fig — (A-D) Show that grh-GAL4 driven expression of UAS-nls-GFP closely correlate with Grh protein expression in abdominal and terminal NBs in early L2 (A), late L2 (B), early L3 (C) and mid L3 stages (D). (E-G) Shows that compared to wild type, grimA6C-reaper17 homozygous double mutants and MM3/M22 transheterozygotes show a block of Dsx-negative NB apoptosis in late L3 female VNCs. (H-J) Shows the expression of 717-lacZ in A8-A10 NBs in female VNCs in mid L2 (H), early L3 (I) and mid L3 (J) stages. (K) Graph showing the quantitation of lacZ intensity in A8-A10 NBs across different stages. Panels “E-G” are single confocal sections; rest all panels are partial z-projects. Yellow arrowheads indicate A8-A10 NBs. White arrowheads in panels “A-D” show abdominal NBs and in panels “F and G” indicate Dsx-positive NBs. Scale bars are 10μm. Graph shows mean±s.d. Significance (P-value) is from two-tailed Student's unpaired t-test. (TIF) [file pgen.1008976.s004.tif]

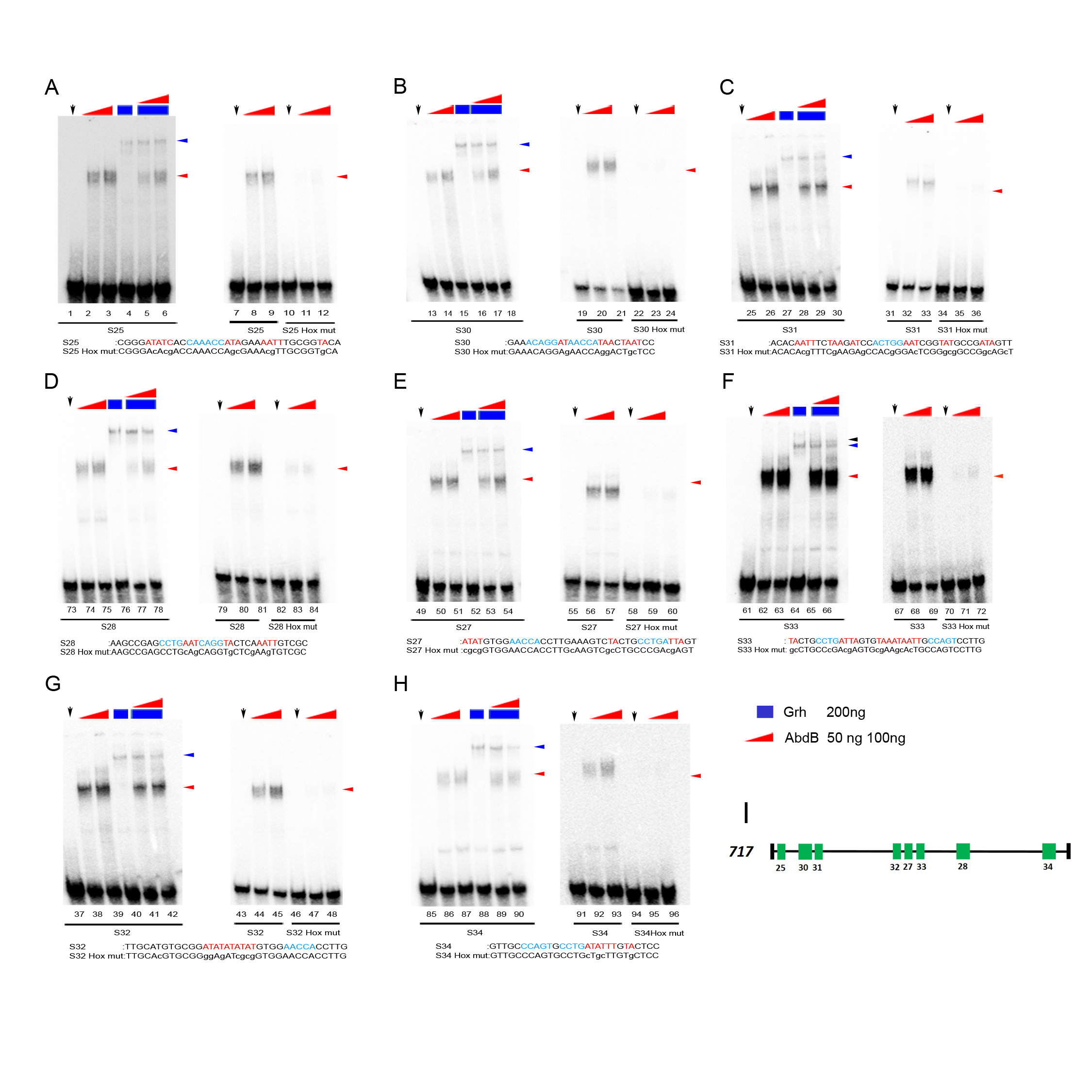

Supplement: S5 Fig — (A-I) EMSA autoradiogram for Abd-B, Grh and Abd-B-Grh binding on oligonucleotides for maintenance motifs-25 (A), 27 (E), 28 (D), 30 (B), 31 (C), 32 (G), 33 (F) and 34 (H) of 717bp enhancer are shown. Wild type and Hox mutant oligonucleotide sequences used are shown at the bottom of each gel. Hox and Grh binding sequences are colour coded in red and blue respectively. Mutations are shown in small case. Only motif-33 (F) shows Abd-B-Grh-DNA complex formation. (I) Shows a schematic of 717bp enhancer showing the relative position of various binding motifs. Proteins added to a specific lane are shown at the top of each lane. Lane with free probes are indicated by downward facing black arrows. EMSA indicate that Abd-A binding sites are also capable of binding Abd-B. Blue rectangles indicate a constant concentration of 200ng for Grh protein. An increasing concentration of 50ng and 100ng of Hox are indicated by red right triangles. Red arrow heads on the gels indicate Hox-DNA complex; while blue arrow heads indicate the Grh-DNA complex. Black arrowhead in panel “F” indicates Grh-Abd-B-DNA complex. (TIF) [file pgen.1008976.s005.tif]

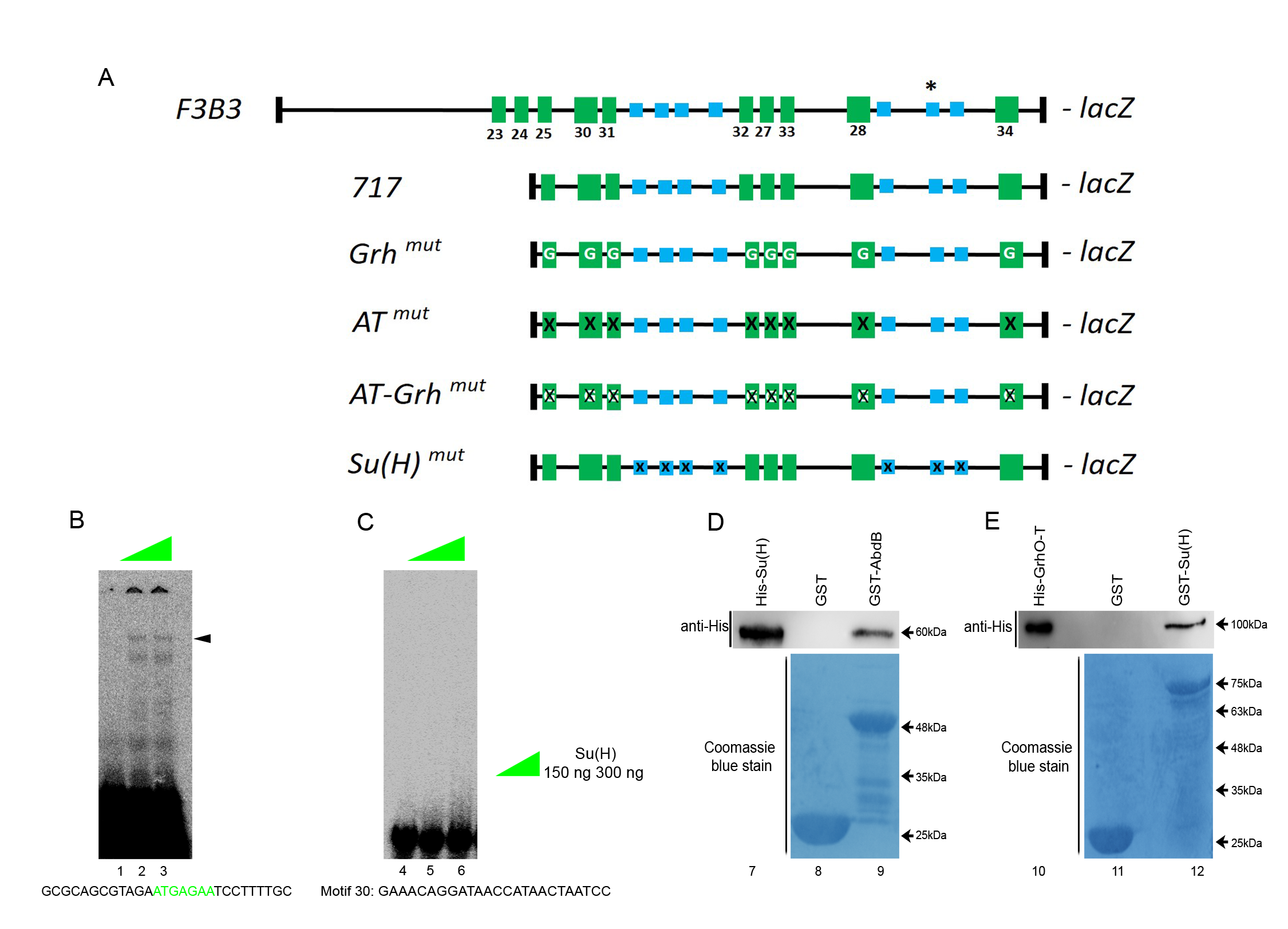

Supplement: S6 Fig — (A) Shows schematic of 1Kb F3B3-lacZ, 717-lacZ and its mutant versions Grhmutant-lacZ, ATmutant-lacZ, AT-Grhmutant-lacZ and Su(H)mutant-lacZ (used in Fig 5 of the main text). Motifs with Grh binding sites are shown as green rectangles (if they have a single Grh binding site) and as green squares (if they have two Grh binding sites). Su(H) binding sites are indicated as blue squares and asterisk indicates the Su(H) binding site for which EMSA is shown in (B). Black crosses indicate mutagenesis of AT rich and Su(H) sites. “G” in white font within green boxes indicate mutation of just Grh binding sites within these motifs. (B) Shows EMSA autoradiogram for Su(H) binding site indicated by asterisks on 717bp enhancer. Out of seven sites shown on the schematic only one site (indicated by asterisk) showed an in vitro binding by Su(H) (indicated by black arrowhead). (C) EMSA autoradiogram showing that Su(H) does not bind on motif-30 which has Hox-Exd-Grh binding sites, lane-1 and 4 show free probes. Right triangle on top of EMSA indicates an increasing concentration of 150 and 300 ng of Su(H) protein. Sequence of oligonucleotides is shown at the bottom of EMSA. (D) Western blot showing in-vitro pulldown assay for bacterially expressed GST-tagged Abd-B (but not GST alone) is able to pull down His-tagged Su(H) (lane-8 vs 9). (E) Western blot showing in-vitro pulldown assay for bacterially expressed GST-tagged Su(H) (but not GST alone) is able to pull down His-tagged Grh (lane-11 vs 12). Coomassie Blue depicts almost equal loading of the GST-tagged protein samples in D and E. (TIF) [file pgen.1008976.s006.tif]

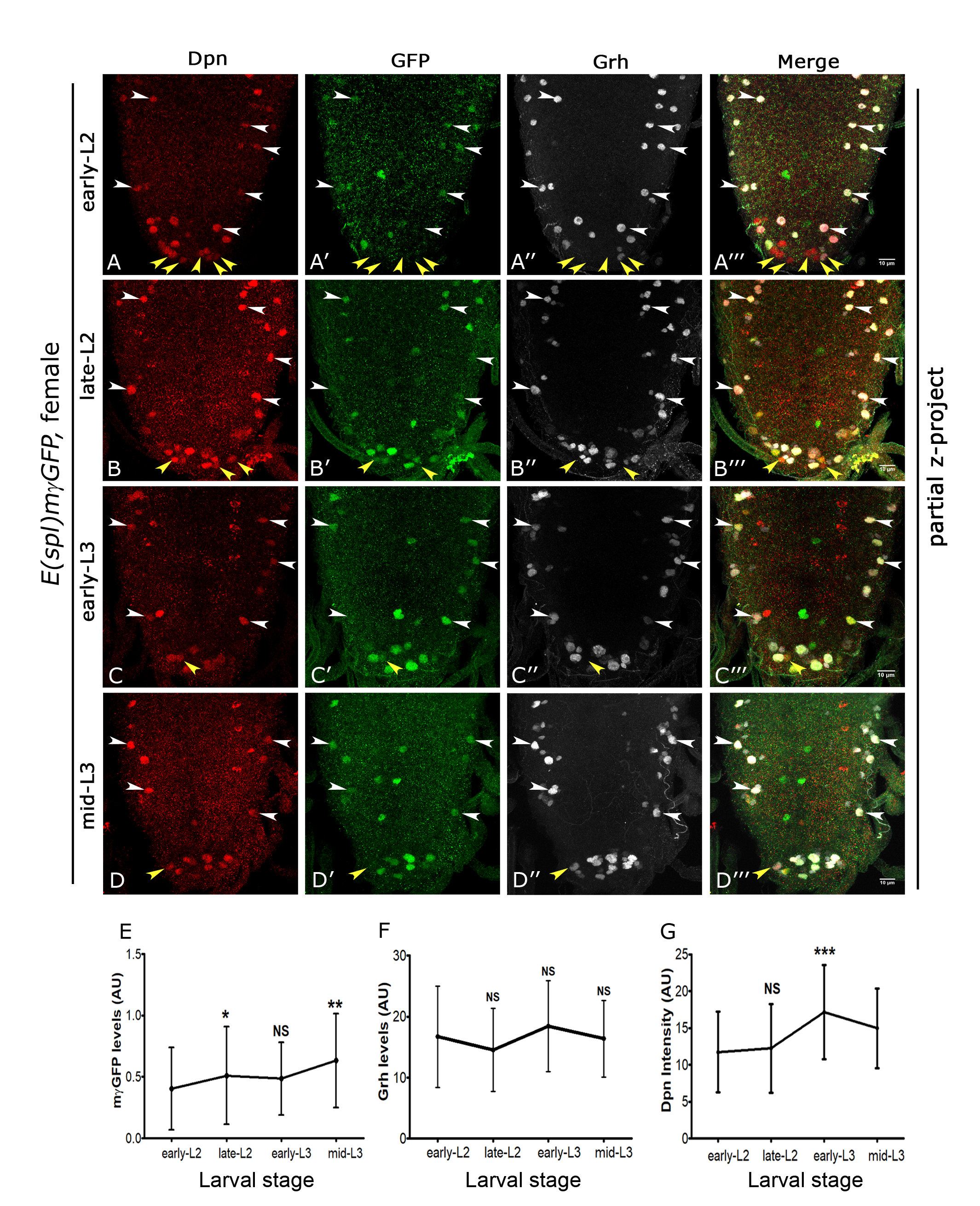

Supplement: S7 Fig — (A-D) Show expression of Grh and E(spl)mγ-GFP across early L2 (A), late L2 (B), early L3 (C) and mid L3 stages (D) in abdominal and terminal NBs of female VNCs. (E-G) Show graphs depicting quantitation of E(spl)mγ-GFP (E), Grh (F), and control Dpn staining (G) intensities across different stages. Graphs indicate that expression of Grh and E(spl)mγ-GFP is mostly constant across different stages in abdominal NBs. Slight but significant difference in E(spl)mγ-GFP expression is seen from early L3 to mid L3 stage. Partial z-projects are shown for A-D to show both abdominal and terminal NBs (indicated by white and yellow arrowheads respectively). Scale bars are 10μm. Graph shows mean±s.d. Significance (P-value) is from two-tailed Student's paired t-test. (TIF) [file pgen.1008976.s007.tif]

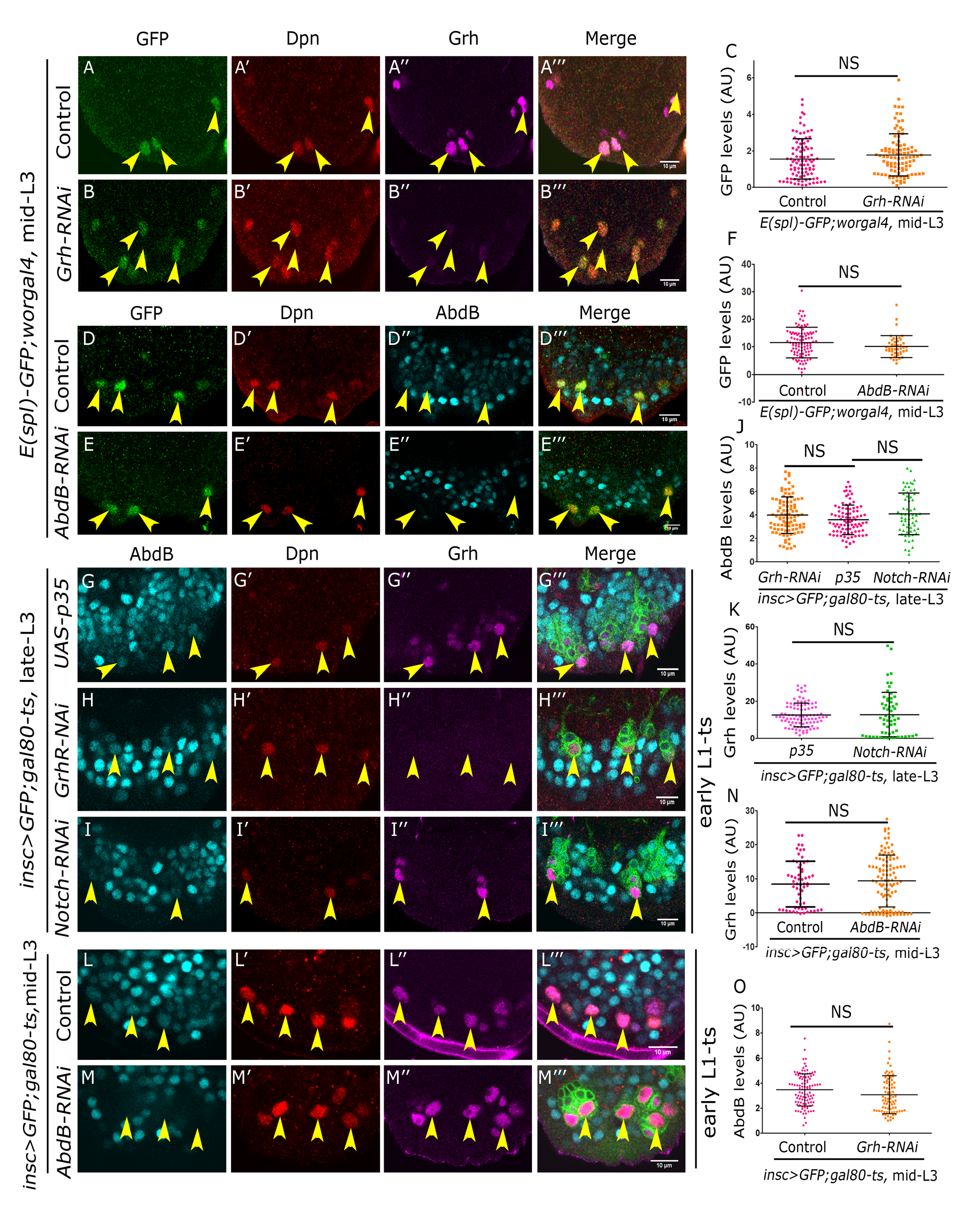

Supplement: S8 Fig — (A-B, D-E) Show that compared to A8-A10 NBs in control VNCs, knockdown of Grh (A-B) and Abd-B (D-E) does not affect E(spl)mγ-GFP expression in mid L3 stage VNCs. (C, F) Graphs showing quantitation of E(spl)mγ-GFP fluorescence in A8-A10 NBs of control VNCs versus Grh knockdown (C) and Abd-B knockdown (F). (G-I) Show that compared to p35 expressing A8-A10 NBs, knockdown for Grh (H) or Notch (I) (TS, S1A Fig) does not affect expression of Abd-B, or Abd-B and Grh respectively. (J) Shows the graph comparing the levels of Abd-B in A8-A10 NBs at late L3 stage in p35 expressing VNCs versus Notch or Grh knockdown (induced from early L1 stage, TS, S1A Fig). (K) Shows the graph comparing the levels of Grh in A8-A10 NBs at late L3 stage in p35 expressing VNCs versus VNCs with Notch knockdown (induced from early L1 stage, TS, S1A Fig). (L-M) Show that compared to control VNCs knockdown of Abd-B from early L1 stage does not affect the expression of Grh in A8-A10 NBs at mid L3 stage (TS, S1G Fig). (N-O) Show the graphs comparing the levels of Grh (N) and Abd-B (O) in A8-A10 NBs at mid L3 stage in control VNCs versus Abd-B knockdown (N) and for control VNCs versus Grh knockdown (O) (TS, S1F Fig). These results indicate that unlike in A3-A7 segments Abd-B, Grh and Notch does not show any cross regulation in A8-A10 segments. Both male and female VNCs were used in these experiments. Yellow arrowheads indicate A8-A10 NBs. Scale bars are 10μm. All images are single confocal sections. Graph shows mean±s.d. Significance (P-value) is from two-tailed Student's unpaired t-test. (TIF) [file pgen.1008976.s008.tif]

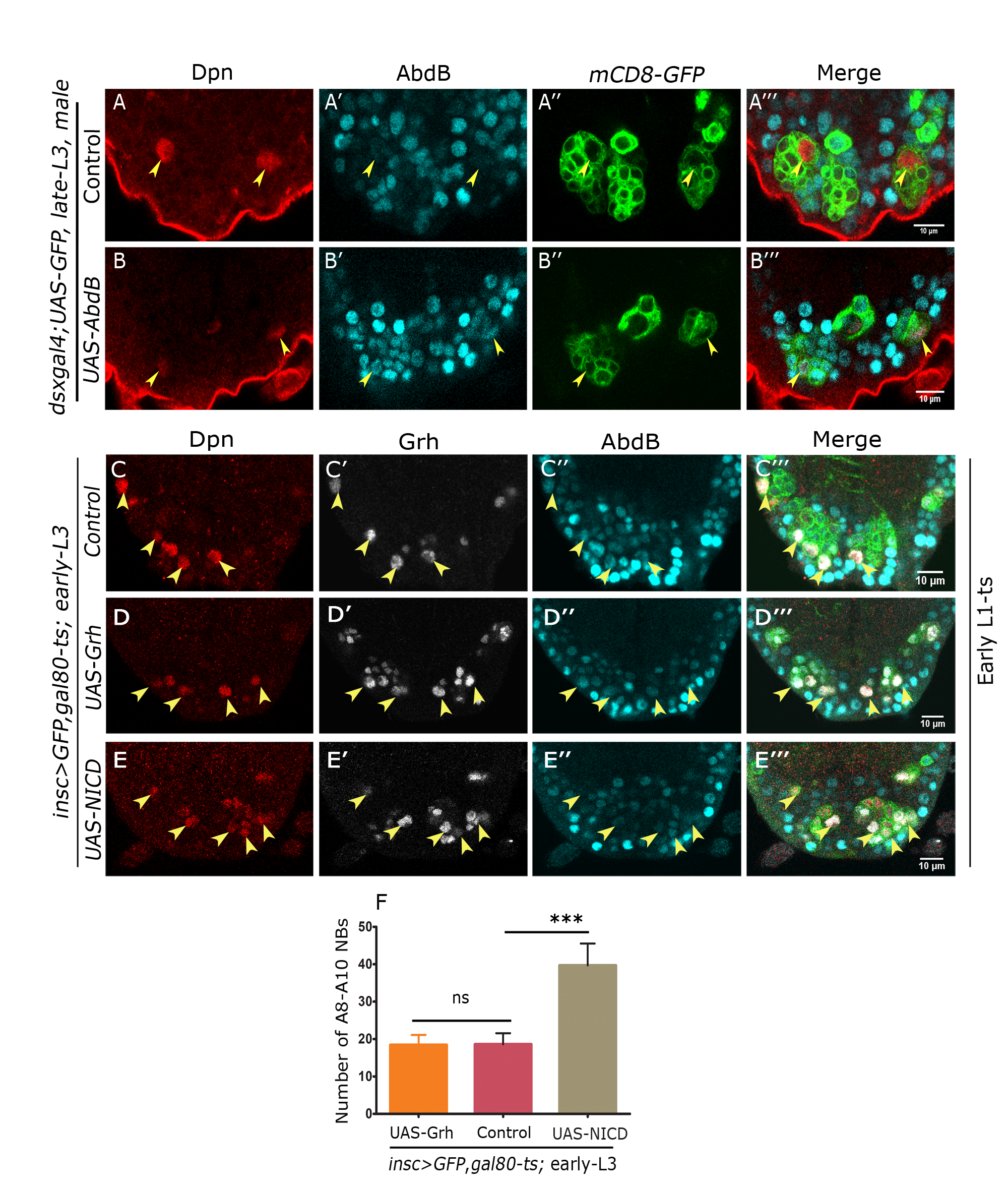

Supplement: S9 Fig — (A-B) Show that compared to control male VNC overexpressing GFP (A), overexpression of Abd-B in Dsx-positive NBs does not result in their apoptosis, indicating that Dsx-positive NBs in males are refractory to apoptosis induced by Abd-B. (C-E) Show that compared to controls (C) overexpression of Grh (D) or NICD (E) from early L1 stage (TS, S1A Fig) does not advance Dsx-negative NB apoptosis to an earlier time point of early L3 stage. (F) Shows a graph comparing the number of surviving A8-A10 NBs at early L3 stage for Grh and NICD overexpression compared to controls. Male VNCs are shown. Yellow arrowheads indicate A8-A10 NBs. Scale bars are 10μm. All images are single confocal sections. Graph shows mean±s.d. Significance (P-value) is from two-tailed Student's unpaired t-test. (TIF) [file pgen.1008976.s009.tif]

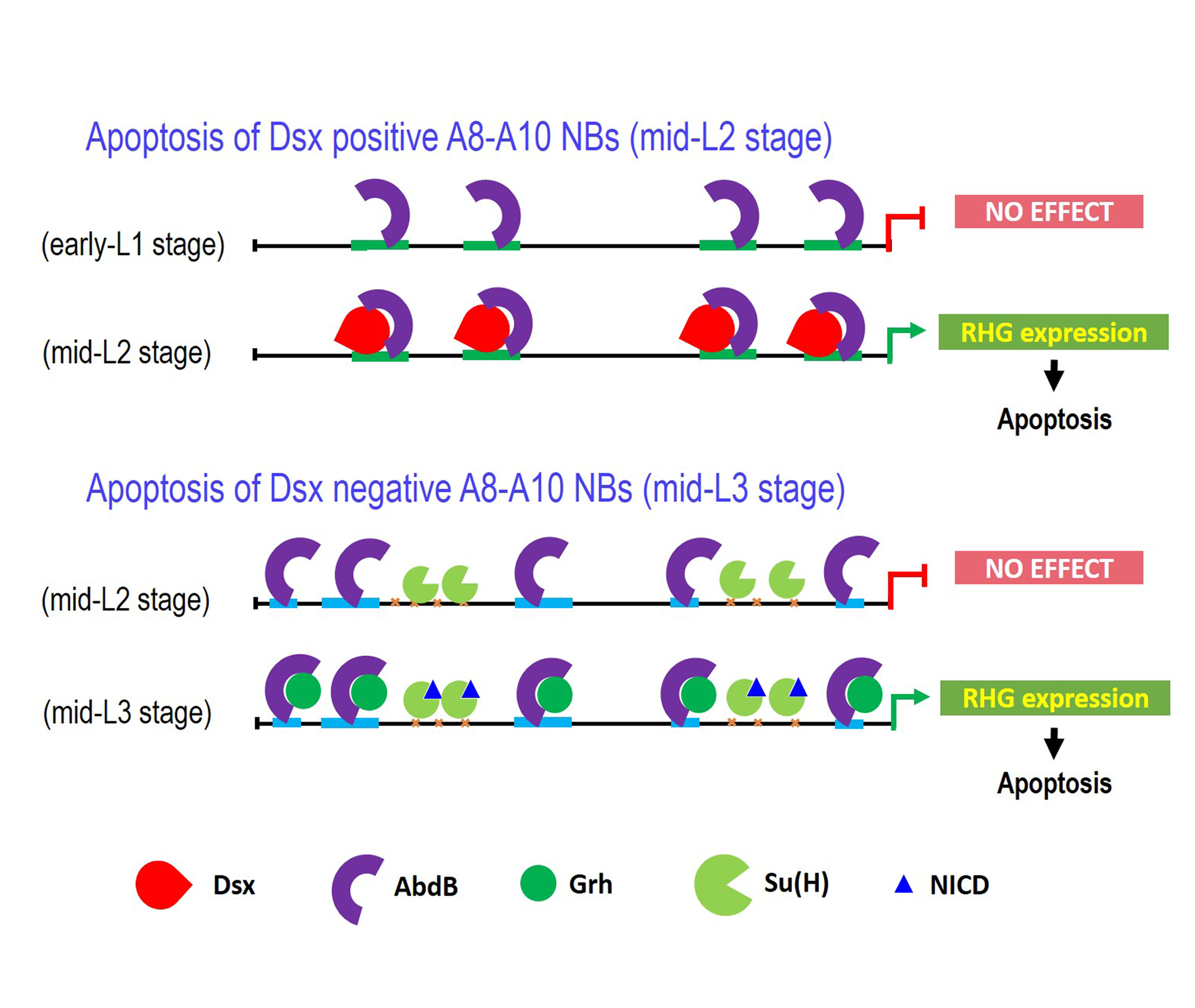

Supplement: S10 Fig — Dsx-positive NBs die at mid L2 stage, wherein Abd-B (shown in purple) and DsxF (shown in red) cooperate to activate apoptotic enhancer to cause female specific apoptosis. This happens on a specific set of Abd-B-Dsx binding motifs (shown by green boxes) on the enhancer (shown by black line). While Dsx-negative NBs undergo apoptosis in mid L3 stage relying on presence of Abd-B, Grh (shown by dark green filled circle) and Notch (shown in dark blue triangle) using a completely different set of binding motifs (shown as light blue boxes). These motifs are required for maintenance of the apoptotic enhancer in Dsx-negative NBs. Abdominal NBs in A3-A7 segments also rely on the same motifs (shown as light blue boxes) for maintaining the activity of the enhancer in abdominal NBs. (TIF) [file pgen.1008976.s010.tif]
