# Supplementary material for: Sequential activation of Notch and Grainyhead gives apoptotic competence to Abdominal-B expressing larval neuroblasts in Drosophila Central nervous system
Source: PLoS Genet. 2020 Aug 31;16(8):e1008976. doi: 10.1371/journal.pgen.1008976 (PMC7485976; doi:10.1371/journal.pgen.1008976)
Supplement: S1 Data — (DOCX) [file pgen.1008976.s011.docx]

**Number and time of death of Dsx-negative NBs in A8-A10 segments of VNCs**

The temporal window of Dsx-negative NB apoptosis was identified using female larvae which over expressed p35 in A8-A10 NBs at different stages of larval development (early L1, 24-28 hrs AEL; mid L2, 60-64 hrs AEL; early L3, 84-88 hrs AEL; mid L3, 96-100 hrs and 100-104 hrs AEL, TS, S1A-S1E Fig, see materials and methods for details). These larvae were dissected in the late L3 stage (Fig 1F). We could recover majority of the NBs in female VNCs when p35 was expressed from early L1 (19.5+/- 2.12; n=15 VNCs, N=3, TS, S1A Fig), mid L2 (16.75+/- 3.40; n=12 VNCs, N=3, TS, S1E Fig), early L3 stages (15.87+/- 3.94; n=8 VNCs, N=3, TS, S1C Fig). In case of temperature shift for mid L3 stages (4.5+/- 2.67; n=16 VNCs, N=4, TS, S1D Fig) we could recover only a quarter of the total population and this number was reduced to only 2 or less per VNC in cases where p35 was expressed in NBs from a time later than 96-100 hrs AEL (1.8+/-1.09; n=5, VNCs, N=3, TS, S1D Fig). Since Dsx-positive NBs are known to undergo death at mid L2 stage [[1](#_ENREF_1)]. These results suggested that Dsx-negative NBs (like A3-A7 NBs) undergo apoptosis in the early-mid L3 stage.

**Su(H) binding on 717 enhancer**

We had earlier identified 7 Su(H) binding sites within 717 bp subfragment of 1Kb apoptotic enhancer. These sites were close variants of the consensus Su(H) binding sequence of RTGRGAR [[2](#_ENREF_2),[3](#_ENREF_3)]. We tested all these sites for the binding of Su(H) protein by EMSA. We found that except one site (indicated by asterisks in the S6A Fig) which showed us a Su(H) binding (shown in S6B Fig), none of the other binding sites showed any Su(H) binding in EMSA. Next we tested *enhancer-lacZ* mutagenized for a single Su(H) binding site, for its capacity to maintain enhancer expression in late L3 stage. We observed that mutagenesis of just one Su(H) binding site did not affect the maintenance capacity of the apoptotic enhancer in abdominal NBs. Results implied that maintenance capacity of the enhancer relies on all the 7 identified Su(H) binding sites, and perhaps also on additional non-consensus Su(H) binding sites which are yet to be identified on the enhancer.

Next, we tested Su(H) for its interaction with Hox gene AbdB as well as Grh. We observed that GST-tagged AbdB (but not GST alone) was able to pull down His-tagged Su(H) (lane-8 vs 9, S6D Fig). Similarly, GST-tagged Su(H) (but not GST alone) was found to be capable of interacting with His-tagged Grh (lane-11 vs 12). But since all the identified Su(H) binding sites were more than 20 bp away [[4](#_ENREF_4)] from the validated AT-Grh binding motifs, therefore the significance of these interactions could not be tested.

The results suggested that there are likely to be other non-consensus Su(H) binding sites on the enhancer which may play a role in its activity maintenance. Therefore, it is a possibility that some of these non-consensus Su(H) binding sites may be in closer vicinity of AT-Grh motifs. To this end, we tested motif-30 (one of the good binders of Hox-Exd-Grh) on 717bp enhancer [[3](#_ENREF_3)], for Su(H) binding by EMSA. We found that Su(H) did not show any binding on motif-30 (S6C Fig). We believe an unbiased scanning of the enhancer by EMSA for Su(H) binding sites will be required to identify additional Su(H) binding sites.

**AbdB, Grh and Notch do not cross regulate each other in A8-A10 NBs**

All below mentioned cross regulation experiments are done with mixed populations of male and female larval VNCs. Therefore, 20% of the cells analysed will be Dsx-positive and rest 80% of the cells will be Dsx-negative.

Grh and AbdB do not cross regulate each other in A8-A10 NBs.

To check if Grh and AbdB cross regulate each other, we started out by knocking down Grh in A8-A10 NBs using RNAi from early L1 stage (TS, S1A Fig), and the expression of AbdB was monitored in late L3 stage, and compared to p35 expressing A8-A10 NBs. We observed no significant change in expression of AbdB in case of *grh-RNAi* (3.07+/- 1.51, n=83 NBs, N=3) compared to p35 expressing NBs (3.46+/- 1.28, n=102 NBs, N=3) (S8G-S8H Fig and S8J Fig). Similarly, *AbdB-RNAi* was carried out and Grh was monitored prior to cell death in A8-A10 NBs (TS, S1F Fig). Here also we observed that Grh expression was unchanged in case of *AbdB-RNAi* (8.62+/- 6.65, n=59 NBs, N=3) compared to A8-A10 NBs in control VNCs (9.26+/- 7.52, n=106 NBs VNCs, N=3) (S8L-S8N Fig).

AbdB and Notch do not cross regulate each other in A8-A10 NBs

Next, we wanted to test if AbdB expression was regulated by Notch activity. For this we knocked down *Notch* gene and tested the expression of AbdB (TS, S1A Fig). We found that AbdB expression was unaffected in A8-A10 NBs in case of *Notch-RNAi* (3.38+/-2.24, n=83 NBs, N=3) compared to p35 expressing NBs (3.46+/-1.28, n=102 NBs, N=3) (S8G Fig, S8I Fig and S8J Fig). A converse cross regulation of Notch signalling by AbdB was also tested, wherein E(spl)mγ-GFP expression was compared in A8-A10 NBs of control versus AbdB knockdown at mid L3 stage. We observed that compared to controls (11.58+/-5.60, n=101 NBs, N=2) the E(spl)mγ-GFP expression was unaffected in case of AbdB knockdown (10.16+/-3.96, n=46 NBs, N=2) (S8D-S8F Fig).

Notch and Grh do not cross regulate each other in A8-A10 NBs

To check if there was any cross regulation between Notch and Grh, we knocked down Notch by RNAi from early L1 stage (S1A Fig) and monitored its effect in late L3 stage. We found that Grh expression in A8-A10 NBs was unaffected in case of Notch knockdown (12.73+/-11.99, n=76 NBs, N=3) when compared to p35 expressing NBs (12.51+/-6.34, n=95 NBs, N=3) (S8G Fig, S8I Fig and S8K Fig). Conversely knockdown of Grh did not have any effect on E(spl)mγ-GFP expression (1.76+/-1.12, n=100 NBs, N=2) compared to controls in mid L3 stage (1.54+/-1.10, n=95 NBs, N=2; S8A-S8C Fig).

**Genotypes analysed**

**Fig 1**

Fig 1B:

*UAS-Dcr2/Y; inscGAL4, UAS-mCD8-GFP/SM6 CyO; gal80-ts/gal80-ts*

*UAS-Dcr2/w; inscGAL4, UAS-mCD8-GFP/SM6 CyO; gal80-ts/gal80-ts*

*UAS-Dcr2/w; inscGAL4, UAS-mCD8-GFP/+; gal80-ts/ UAS-p35*

*UAS-Dcr2/Y; inscGAL4, UAS-mCD8-GFP/+; gal80-ts/ UAS-p35*

Fig 1C-C’’: *UAS-Dcr2/w; inscGAL4, UAS-mCD8-GFP/SM6 CyO; gal80-ts/gal80-ts*

Fig 1D-D’’: *UAS-Dcr2/w; inscGAL4, UAS-mCD8-GFP/SM6 Cyo; gal80-ts/ UAS-p35*

Fig 1E-E’’: *UAS-Dcr2/w; inscGAL4, UAS-mCD8-GFP/+; gal80-ts/ UAS-AbdB-RNAi*

Fig 1F: *UAS-Dcr2/w; inscGAL4, UAS-mCD8-GFP/+; gal80-ts/ UAS-p35*

Fig 1G:

*UAS-Dcr2/w; inscGAL4, UAS-mCD8-GFP/UAS-abdA-RNAi; gal80-ts/ +*

*UAS-Dcr2/w; inscGAL4, UAS-mCD8-GFP/+; gal80-ts/ UAS-AbdB-RNAi*

*UAS-Dcr2/w; inscGAL4, UAS-mCD8-GFP/SM6 CyO; gal80-ts/gal80-ts*

*UAS-Dcr2/w; inscGAL4, UAS-mCD8-GFP/UAS-exd-RNAi; gal80-ts/+*

*UAS-Dcr2/w; inscGAL4, UAS-mCD8-GFP/+ ; gal80-ts/UAS-hth-RNAi*

Fig 1H: *Canton S*

Fig 1I-I’’: *elavGAL4, UAS-mCD8-GFP, hsFLP w/Y; FRT82B tub-GAL80/ FRT82B-AbdB-M1*

Fig 1J-J’: *hsflp, FRT19A, tub-GAL80/FRT19A-exd^1^; tubGAL4, UAS-mCD8-GFP/+*

Fig 1K-K’: *elavGAL4, UAS-mCD8-GFP, hsFLP w/Y; FRT82B tub-GAL80/ FRT82B-hth^P2^*

**Fig 2**

Fig 2A-A’’’: *Canton S*

Fig 2B-B’’: *UAS-Dcr2/w; inscGAL4, UAS-mCD8-GFP/SM6 CyO; gal80-ts/gal80-ts*

Fig 2C-C’’: *UAS-Dcr2/w; inscGAL4, UAS-mCD8-GFP/UAS-grh-RNAi; gal80-ts/+*

Fig 2D-D’’: *UAS-Dcr2/w; inscGAL4, UAS-mCD8-GFP/+; gal80-ts/ UAS-Notch-RNAi*

Fig 2E:

*UAS-Dcr2/w; inscGAL4, UAS-mCD8-GFP/SM6 CyO; gal80-ts/gal80-ts*

*UAS-Dcr2/w; inscGAL4, UAS-mCD8-GFP/UAS-grh-RNAi; gal80-ts/+*

*UAS-Dcr2/w; inscGAL4, UAS-mCD8-GFP/+; gal80-ts/ UAS-Notch-RNAi*

*w/w; grh^B37^; grh^Df^*

Fig 2F-F’’’: *w/Y; Notch-GFP.FLAG / Notch-GFP.FLAG*

Fig 2G-G’: *UAS-Dcr2/w; inscGAL4, UAS-mCD8-GFP/+; gal80-ts/ UAS-Dl-RNAi*

Fig 2H-H’: *w/w; elavGAL4, UAS-mCD8-GFP/+; UAS-Dl-RNAi/+*

Fig 2I-I’’’’: *UAS-Dcr2/w; inscGAL4, UAS-mCD8-GFP/+; gal80-ts/ UAS-p35*

Fig 2J:

*UAS-Dcr2/+; +/+; repoGAL4, UAS-GFP/UAS-Dl-RNAi*

*w/w; +/+; grhGAL4/UAS-Dl-RNAi*

*UAS-Dcr2/w; inscGAL4, UAS-mCD8-GFP/+; gal80-ts/ UAS-Dl-RNAi*

*w/w; elavGAL4, UAS-mCD8-GFP/+; UAS-Dl-RNAi/+*

**Fig 3**

Fig 3B:

*w/w; +/+; grim^A6C^-rpr^17^/ grim^A6C^-rpr^17^*

*w/w; +/+; grim^A6C^/ grim^A6C^*

*w/w;+/+; rpr^17^/ rpr^17^*

*Canton S*

*w/w; +/+; M22/MM3*

*w/w; +/+; MM3/MM3*

Fig 3C-C’’: *w/w; +/+; F3B3-lacZ/F3B3-lacZ*

Fig 3D-D’’: *w/w; 717-lacZ/717-lacZ*

Fig 3E-E’’’’: *UAS-Dcr2/w; inscGAL4, UAS-mCD8-GFP/F3B3-lacZ; gal80-ts/ UAS-p35*

Fig 3F-F’’’’: *UAS-Dcr2/w; inscGAL4, UAS-mCD8-GFP/F3B3-lacZ; gal80-ts/ UAS-AbdB-RNAi*

Fig 3G-G’’’’: *UAS-Dcr2/w; inscGAL4, UAS-mCD8-GFP/F3B3-lacZ; gal80-ts/ UAS-Notch-RNAi*

Fig 3H-H’’’’: *UAS-Dcr2/w; inscGAL4, UAS-mCD8-GFP/UAS-grh-RNAi; gal80-ts/ F3B3-lacZ*

Fig 3I:

*UAS-Dcr2/w; inscGAL4, UAS-mCD8-GFP/F3B3-lacZ; gal80-ts/ UAS-p35*

*UAS-Dcr2/w; inscGAL4, UAS-mCD8-GFP/F3B3-lacZ; gal80-ts/ UAS-AbdB-RNAi*

*UAS-Dcr2/w; inscGAL4, UAS-mCD8-GFP/F3B3-lacZ; gal80-ts/ UAS-Notch-RNAi*

*UAS-Dcr2/w; inscGAL4, UAS-mCD8-GFP/UAS-grh-RNAi; gal80-ts/ F3B3-lacZ*

**Fig 4**

Fig 4D: w*/Y; +/+; Δ717/ Δ717*

Fig 4E:

*Canton S*

*w/w; +/+; Δ717/ Δ717*

w*/Y; +/+; Δ717/ Δ717*

**Fig 5**

Fig 5A-A’’’: *UAS-Dcr2/w; inscGAL4, UAS-mCD8-GFP/717-lacZ; gal80-ts/ UAS-p35*

Fig 5B-B’’’: *UAS-Dcr2/w; inscGAL4, UAS-mCD8-GFP/Grh^mutant^-lacZ; gal80-ts/ UAS-p35*

Fig 5C-C’’’: *UAS-Dcr2/w; inscGAL4, UAS-mCD8-GFP/AT^mutant^-lacZ; gal80-ts/ UAS-p35*

Fig 5D-D’’’: *UAS-Dcr2/w; inscGAL4, UAS-mCD8-GFP/AT-Grh^mutant^-lacZ; gal80-ts/ UAS-p35*

Fig 5E-E’’’: *UAS-Dcr2/w; inscGAL4, UAS-mCD8-GFP/Su(H)^mutant^-lacZ; gal80-ts/ UAS-p35*

Fig 5F-G:

*UAS-Dcr2/w; inscGAL4, UAS-mCD8-GFP/717-lacZ; gal80-ts/ UAS-p35*

*UAS-Dcr2/w; inscGAL4, UAS-mCD8-GFP/Grh^mutant^-lacZ; gal80-ts/ UAS-p35*

*UAS-Dcr2/w; inscGAL4, UAS-mCD8-GFP/AT^mutant^-lacZ; gal80-ts/ UAS-p35*

*UAS-Dcr2/w; inscGAL4, UAS-mCD8-GFP/AT-Grh^mutant^-lacZ; gal80-ts/ UAS-p35*

*UAS-Dcr2/w; inscGAL4, UAS-mCD8-GFP/Su(H)^mutant^-lacZ; gal80-ts/ UAS-p35*

**Fig 6**

Fig 6A-D: *w/w; E(spl)mγ-GFP/SM6 CyO*

**Fig 7**

Fig 7A-7B:

*Canton S male*

*UAS-Dcr2/Y; inscGAL4, UAS-mCD8-GFP/UAS-AbdB; gal80-ts/ +*

*UAS-Dcr2/Y; inscGAL4, UAS-mCD8-GFP/UAS-AbdB; gal80-ts/ UAS-Notch-RNAi*

*UAS-Dcr2/Y; inscGAL4, UAS-mCD8-GFP/UAS-AbdB, UAS-grh-RNAi; gal80-ts/ +*

Fig 7C-C’’: *Canton S male*

Fig 7D-D’’’: *UAS-Dcr2/Y; inscGAL4, UAS-mCD8-GFP/UAS-AbdB; gal80-ts/ +*

Fig 7E-E’’’: *UAS-Dcr2/Y; inscGAL4, UAS-mCD8-GFP/UAS-AbdB; gal80-ts/ UAS-Notch-RNAi*

Fig 7F-F’’’: *UAS-Dcr2/Y; inscGAL4, UAS-mCD8-GFP/UAS-AbdB, UAS-grh-RNAi; gal80-ts/ +*

**Supporting Figures**

**S2 Fig**

S2A-S2C: *Canton S females*

S2D-S2D’’: *UAS-Dcr2/w; inscGAL4, UAS-mCD8-GFP/+; gal80-ts/ UAS-p35*

S2E-S2E’’: *UAS-Dcr2/w; inscGAL4, UAS-mCD8-GFP/+; gal80-ts/ UAS-AbdB-RNAi*

S2F-S2F’’: *UAS-Dcr2/w; inscGAL4, UAS-mCD8-GFP/UAS-abdA-RNAi; gal80-ts/ +*

S2G:

*UAS-Dcr2/w; inscGAL4, UAS-mCD8-GFP/+ ; gal80-ts/ +*

*UAS-Dcr2/w; inscGAL4, UAS-mCD8-GFP/UAS-abdA-RNAi; gal80-ts/ +*

**S3 Fig**

S3A-S3E: *w/w; +/+; Notch-GFP.FLAG/+*

S3F-S3F’’: *UAS-Dcr2/w; inscGAL4, UAS-mCD8-GFP/+; gal80-ts/ UAS-p35*

S3G-S3G’’: *UAS-Dcr2/w; inscGAL4, UAS-mCD8-GFP/+; gal80-ts/ UAS-Dl-RNAi*

**S4 Fig**

S4A-S4D: *w/w; +/+; grhGAL4, UAS-nls-GFP/+*

S4E-S4E’’: *Canton S*

S4F-S4F’’: *w/w; +/+; grim^A6C^-rpr^17^/ grim^A6C^-rpr^17^*

S4G-S4G’’: *w/w; +/+; MM3/M22*

S4H-S4K: *w/w; 717-lacZ/717-lacZ*

**S7 Fig**

S6A-S6D: *w/w; E(spl)mγ-GFP/SM6, CyO*

**S8 Fig**

S8A-S8A’’’: *w/w; E(spl)mγ-GFP/+; worGAL4/+*

S8B-S8B’’’: *w/w; E(spl)mγ-GFP/UAS-grh-RNAi; worGAL4/+*

S8C:

*w/w; E(spl)mγ-GFP/+; worGAL4/+*

*w/w; E(spl)mγ-GFP/UAS-Grh-RNAi; worGAL4/+*

S8D-S8D’’’: *w/w; E(spl)mγ-GFP/+; worGAL4/+*

S8E-S8E’’’: *w/w; E(spl)mγ-GFP/+; worGAL4/UAS-AbdB-RNAi*

S8F:

*w/w; E(spl)mγ-GFP/+; worGAL4/+*

*w/w; E(spl)mγ-GFP/+; worGAL4/UAS-AbdB-RNAi*

S8G-S8G’’’: *UAS-Dcr2/w; inscGAL4, UAS-mCD8-GFP/+; gal80-ts/ UAS-p35*

S8H-S8H’’’: *UAS-Dcr2/w; inscGAL4, UAS-mCD8-GFP/UAS-grh-RNAi; gal80-ts/+*

S8I-S8I’’’: *UAS-Dcr2/w; inscGAL4, UAS-mCD8-GFP/+; gal80-ts/ UAS-Notch-RNAi*

S8J:

*UAS-Dcr2/w; inscGAL4, UAS-mCD8-GFP/+; gal80-ts/ UAS-p35*

*UAS-Dcr2/w; inscGAL4, UAS-mCD8-GFP/UAS-grh-RNAi; gal80-ts/+*

*UAS-Dcr2/w; inscGAL4, UAS-mCD8-GFP/+; gal80-ts/ UAS-Notch-RNAi*

S8K:

*UAS-Dcr2/w; inscGAL4, UAS-mCD8-GFP/+; gal80-ts/ UAS-p35*

*UAS-Dcr2/w; inscGAL4, UAS-mCD8-GFP/+; gal80-ts/ UAS-Notch-RNAi*

S8L-L’’’: *UAS-Dcr2/w; inscGAL4, UAS-mCD8-GFP/+; gal80-ts/ +*

S8M-S8M’’’: *UAS-Dcr2/w; inscGAL4, UAS-mCD8-GFP/+; gal80-ts/ UAS-AbdB-RNAi*

S8N:

*UAS-Dcr2/w; inscGAL4, UAS-mCD8-GFP/+; gal80-ts/ +*

*UAS-Dcr2/w; inscGAL4, UAS-mCD8-GFP/+; gal80-ts/ UAS-AbdB-RNAi*

S8O:

*UAS-Dcr2/w; inscGAL4, UAS-mCD8-GFP/+; gal80-ts/ +*

*UAS-Dcr2/w; inscGAL4, UAS-mCD8-GFP/UAS-grh-RNAi; gal80-ts/ +*

**S9 Fig**

S9A-S9A’’’: *w/w; UAS-mCD8-GFP/SM6 CyO; dsxGAL4/+*

S9B-S9B’’’: *w/w; UAS-mCD8-GFP/UAS-AbdB; dsxGAL4/+*

S9C-S9C’’’: *UAS-Dcr2/w; inscGAL4, UAS-mCD8-GFP/+; gal80-ts/ +*

S9D-S9D’’’: *UAS-Dcr2/w; inscGAL4, UAS-mCD8-GFP/UAS-grh; gal80-ts/ +*

S9E-S9E’’’: *UAS-Dcr2/w; inscGAL4, UAS-mCD8-GFP/UAS-NICD; gal80-ts/ +*

S9F:

*UAS-Dcr2/w; inscGAL4, UAS-mCD8-GFP/+; gal80-ts/ +*

*UAS-Dcr2/w; inscGAL4, UAS-mCD8-GFP/UAS-grh; gal80-ts/ +*

*UAS-Dcr2/w; inscGAL4, UAS-mCD8-GFP/UAS-NICD; gal80-ts/ +*

References:

1. Ghosh N, Bakshi A, Khandelwal R, Rajan SG, Joshi R (2019) The Hox gene Abdominal-B uses Doublesex(F) as a cofactor to promote neuroblast apoptosis in the Drosophila central nervous system. Development 146.

2. Nellesen DT, Lai EC, Posakony JW (1999) Discrete enhancer elements mediate selective responsiveness of enhancer of split complex genes to common transcriptional activators. Dev Biol 213: 33-53.

3. Khandelwal R, Sipani R, Govinda Rajan S, Kumar R, Joshi R (2017) Combinatorial action of Grainyhead, Extradenticle and Notch in regulating Hox mediated apoptosis in Drosophila larval CNS. PLoS Genet 13: e1007043.

4. Sorge S, Ha N, Polychronidou M, Friedrich J, Bezdan D, et al. (2012) The cis-regulatory code of Hox function in Drosophila. EMBO J 31: 3323-3333.
